# Premeiotic deletion of *Eif2s2* causes oocyte arrest at the early diplotene stage and apoptosis in mice

**DOI:** 10.1111/cpr.13718

**Published:** 2024-07-24

**Authors:** Wenjun Zhou, Biao Li, Zhijuan Wang, Shuang Liu, Weiyong Wang, Sihui He, Ye Chen, Xiaodan Zhang, Meijia Zhang

**Affiliations:** ^1^ The Innovation Centre of Ministry of Education for Development and Diseases, the Second Affiliated Hospital, School of Medicine South China University of Technology Guangzhou China; ^2^ Center for Sleep and Circadian Medicine The Affiliated Brain Hospital of Guangzhou Medical University Guangzhou China

## Abstract

Eukaryotic translation initiation factor 2 subunit 2 (EIF2S2), a subunit of the heterotrimeric G protein EIF2, is involved in the initiation of translation. Our findings demonstrate that the depletion of *Eif2s2* in premeiotic germ cells causes oocyte arrest at the pachytene and early diplotene stages at 1 day postpartum (dpp) and 5 dpp, respectively, and eventually leads to oocyte apoptosis and failure of primordial follicle formation. Further studies reveal that *Eif2s2* deletion downregulates homologous recombination‐related and mitochondrial fission‐related protein levels, and upregulates the integrated stress response‐related proteins and mRNA levels. Consistently, *Eif2s2* deletion significantly decreases the expression of dictyate genes and compromises mitochondrial function, characterized by elongated shapes, decreased ATP levels and mtDNA copy number, along with an excessive accumulation of reactive oxygen species (ROS) and mitochondrial superoxide. Furthermore, DNA damage response and proapoptotic protein levels increase, while anti‐apoptotic protein levels decrease in *Eif2s2*‐deleted mice. An increase in oocytes with positive cleaved‐Caspase‐3 and TUNEL signals, alongside reduced Lamin B1 intensity, further indicates oocyte apoptosis. Collectively, *Eif2s2* deletion in premeiotic germ cells causes oocyte meiotic arrest at the early diplotene stage by impairing homologous recombination, and eventually leads to oocyte apoptosis mainly through the downregulation of mitochondrial fission‐related proteins, ROS accumulation and subsequent DNA damage.

## Graphical abstract

Premeiotic deletion of *Eif2s2* impairs the translation of proteins related to homologous recombination and mitochondrial fission. These impairments resulted in oocyte meiotic arrest at the early diplotene stage, and eventually led to the apoptosis of oocytes and the failure of primordial follicle formation.

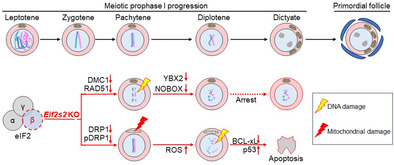

## 1 INTRODUCTION

During mammalian embryonic development, female germ cells reach the ovary and undergo multiple rounds of mitosis to form cysts. Subsequently, the germ cells enter meiosis and go through the leptotene, zygotene and pachytene stages before being arrested at the late diplotene stage (dictyate stage).^1^, ^2^ During meiosis prophase I, a cascade of chromosomal events unfolds, involving the loading meiotic‐specific cohesins onto chromosomes, the synapsis of homologous chromosomes and the generation of crossovers facilitated by meiotic recombination. The initiation of meiotic recombination occurs through SPO11‐induced DNA double‐strand breaks (DSBs), followed by homologous recombination (HR), which are critical for accurate chromosome segregation during meiosis I and for promoting genome diversity.^3^ Some cyst cells (pro‐oocytes) receive cytoplasm and organelles from the other cyst cells (nurse cells) to become oocytes.^4^, ^5^ Meanwhile, somatic cells invade the cysts and envelop oocytes to form nonrenewable primordial follicles. A diminished primordial follicle pool could lead to premature ovarian insufficiency (POI). The process of primordial follicle formation is regulated by several signalling pathways such as Notch, KIT‐PI3K and JNK pathways.^6^, ^7^, ^8^, ^9^ During meiotic prophase I, especially at the dictyate stage, abundant proteins and genes are upregulated in oocytes.^5^, ^10^ In addition, primordial follicle formation was accompanied by massive protein synthesis, which is involved in oogenesis and metabolic processes.^11^ Therefore, the intricate temporal and spatial patterns of protein translation are crucial for primordial follicle formation.

Mutations in the genes encoding eukaryotic translation initiation factors (eIFs) have been reported in POI patients,^12^, ^13^, ^14^ such as *EIF2B2*, *EIF2B4*, *EIF2B5*, eIF1A X‐linked (*EIF1AX*) and eIF4E nuclear import factor 1 (*EIF4ENIF1*). However, the underlying pathogenesis of POI induced by mutations in translation initiation factors remains largely unknown. The germline‐specific isoform eIF4E1B translates maternal mRNAs with GC‐rich elements in 5′UTRs. Deleting *Eif4e1b* in mouse oocytes causes 2‐cell stage arrest by impairing translation of maternal mRNAs critical for oocyte to embryo transition and zygotic genome activation.^15^, ^16^ EIF4ENIF1, an eIF4E binding protein, regulates translation by preventing eIF4E from initiating translation. Haploinsufficiency of *Eif4enif1* leads to subfertility in mice by altering the translation of oocyte mitochondrial fission and fusion proteins.^14^ Mutations in other translation initiation regulators also impair oocyte development and meiotic progression. The mammalian target of rapamycin (mTOR) regulates translation initiation through modulating the phosphorylation of elF4E binding protein 1 and S6 kinase. The deletion of mTOR in oocytes impairs follicular development and oocyte maturation by decreasing the expression of proteins related to mRNA metabolism and actin filament bundle assembly.^17^ RPS26 is a component of the small ribosomal subunit involved in translation initiation. Deletion of *Rps26* leads to the apoptosis of oocytes within preantral follicles by repressing the PI3K/AKT/FOXO3A pathway.^18^ These findings suggest that defects in various translational regulatory proteins lead to diverse reproductive phenotypes.

Eukaryotic initiation factor 2 subunit 2 (EIF2S2), also known as eIF2β, constitutes one of the three subunits of eIF2 that is involved in the formation of a ternary complex alongside Met‐tRNAi and GTP.^19^ Mutations at Ser^2^ and Ser^67^ or the deletion of the polylysine stretches of EIF2S2 leads to downregulated protein synthesis in HEK‐293 T and HeLa cells,^20^, ^21^ respectively. *Eif2s2* deletion in mice causes recessive lethality, and haploinsufficiency of *Eif2s2* attenuated male germ cell proliferation and differentiation.^22^ Studies of mutations in translation initiation factors have mainly focused on follicular development,^14^, ^15^ but their effects on primordial follicle formation remain insufficiently investigated.

Here, we created a premeiotic germ cell‐specific conditional knockout (cKO) mouse by deleting floxed *Eif2s2* alleles using *Stra8*‐Cre. Our results showed that the depletion of *Eif2s2* in premeiotic germ cells impaired the translation of proteins related to HR and mitochondrial fission. These impairments resulted in oocyte meiotic arrest at the early diplotene stage and eventually led to the apoptosis of oocytes and the failure of primordial follicle formation.

## 2 RESULTS

### 2.1 The depletion of eukaryotic translation initiation factor 2 subunit 2 in premeiotic germ cells caused oocytes meiotic arrest and the failure of primordial follicle formation

**FIGURE 1:**
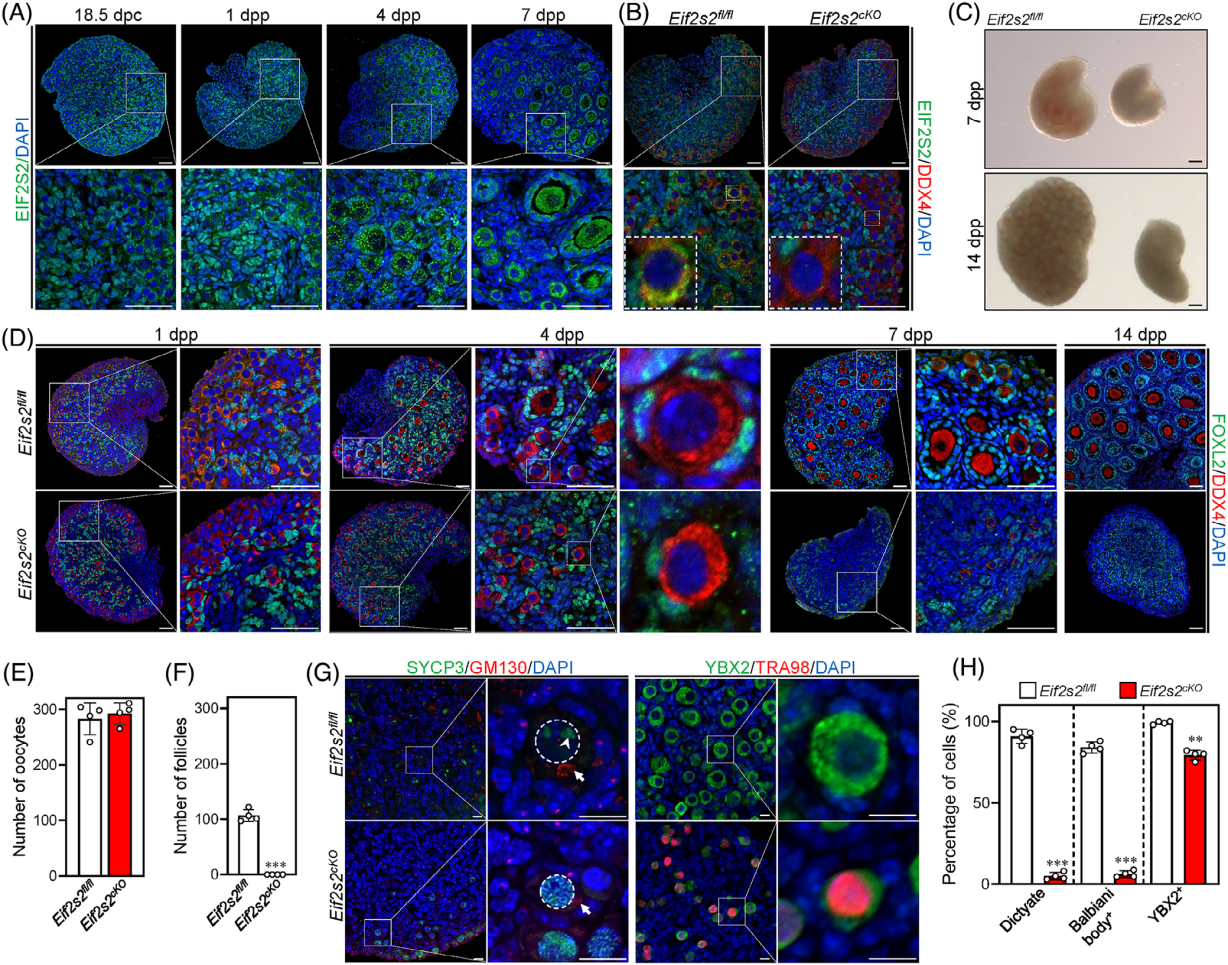
Deletion of *Eif2s2* in premeiotic germ cells causes oocyte premature meiotic arrest and the failure of primordial follicle formation in mice. (A) Representative images of EIF2S2 staining in mouse ovaries at 18.5 days post‐coitum (dpc), 1,4 and 7 days postpartum (dpp). The enlarged views of the boxed area are shown on the bottom side. EIF2S2, green; DAPI, blue. Scale bar: 50 μM. (B) Immunostaining of EIF2S2 and DDX4 in *Eif2s2* ^*fl/fl*^ and *Eif2s2* ^*cKO*^ mouse ovaries. EIF2S2, green; DDX4, red; DAPI, blue. Scale bar: 50 μM. (C) Photographs of ovaries of 7 and14 dpp *Eif2s2* ^*fl/fl*^ and *Eif2s2* ^*cKO*^ mice. Scale bar: 200 μM. (D) Morphological comparison of ovaries of *Eif2s2* ^*fl/fl*^ and *Eif2s2* ^*cKO*^ mice. FOXL2, green; DDX4, red; DAPI, blue. The enlarged views of the boxed area are shown on the right side. Scale bar: 50 μM. (E) Number of oocytes in *Eif2s2* ^*fl/fl*^ and *Eif2s2* ^*cKO*^ ovaries at 1 dpp. *n* = 4. (F) Number of primordial follicles in *Eif2s2* ^*fl/fl*^ and *Eif2s2* ^*cKO*^ ovaries at 4 dpp. *n* = 4. (G) Representative images of SYCP3 (green, left panel) and GM130 (red, left panel), and YBX2 (green, right panel) and TRA98 (red, right panel) double staining in *Eif2s2* ^*fl/fl*^ and *Eif2s2* ^*cKO*^ ovaries at 3 dpp. The dashed circles indicate the nucleus of oocytes. The arrowhead indicates the SYCP3 cluster in dictyate oocytes. The arrow indicates the Balbiani body. (H) Percentage of oocytes reaching the dictyate stage, with Balbiani body, and with positive YBX signals in *Eif2s2* ^*fl/fl*^ and *Eif2s2* ^*cKO*^ ovaries at 4 dpp. *n* = 4. Bars represent the mean ± SD. A two‐sided Student's *t*‐test was performed to determine *p* values (*p < 0.05, **p < 0.01, and ***p < 0.001).

EIF2S2 was mainly expressed in the cytoplasm of both oocytes and somatic cells within mouse ovaries, and the EIF2S2 signal intensity gradually increases from 1 day postpartum (dpp) to 7 dpp in oocytes (Figure 1A). To analyse the physiological function of *Eif2s2* in the oocyte during primordial follicle formation, *Eif2s2* ^*fl/fl*^ mice were cross‐bred with *Stra8‐Cre* mice to obtain *Eif2s2* ^*fl/fl*^; *Stra8‐Cre* (*Eif2s2* ^*cKO*^) mice (Figure S1A). The effective deletion of *Eif2s2* in oocytes was verified through immunofluorescence, qPCR and Western blotting (WB) (Figures 1B and S1B,C). The ovaries from *Eif2s2* ^*cKO*^ mice were dramatically smaller than that from *Eif2s2* ^*fl/fl*^ mice (Figures 1C and S1D,E). Next, we detected the number of oocytes and primordial follicles in newborn ovaries. No significant difference was observed in the number of oocytes between *Eif2s2* ^*fl/fl*^ and *Eif2s2* ^*cKO*^ mice at 1 dpp (Figure 1D,E). In 4‐dpp *Eif2s2* ^*fl/fl*^ mouse ovaries, oocytes were surrounded by several flattened pregranulosa cells and formed primordial follicles. Oocytes in 4‐dpp *Eif2s2* ^*cKO*^ mouse ovaries were smaller than those in *Eif2s2* ^*fl/fl*^ mouse ovaries and were not enveloped closely by pregranulosa cells (Figure 1D,F), suggesting primordial follicle formation failure. Furthermore, the percentages of oocytes contained Balbiani body (GM130 staining, Figure 1G,H) and oocytes reaching the dictyate stage (SYCP3 staining, Figure 1G,H) were significantly decreased in the ovaries of *Eif2s2* ^*cKO*^ mice compared to those of *Eif2s2* ^*fl/fl*^ mice at 4 dpp. The TRA98 signal was remained in the nucleus and the percentage of YBX2 positive oocytes significantly decreased in the ovaries of *Eif2s2* ^*cKO*^ mouse compared to those of *Eif2s2* ^*fl/fl*^ mice (Figure 1G,H), indicating that the meiosis prophase I progression is delayed. Therefore, oocytes from *Eif2s2* ^*cKO*^ mice could not assemble the primordial follicle and underwent apoptosis at 7 dpp (Figure 1D).

### 2.2 *Eif2s2* deletion decreases the levels of proteins related to oocyte growth and meiotic progression and increases the levels of proteins related to stress response

**FIGURE 2:**
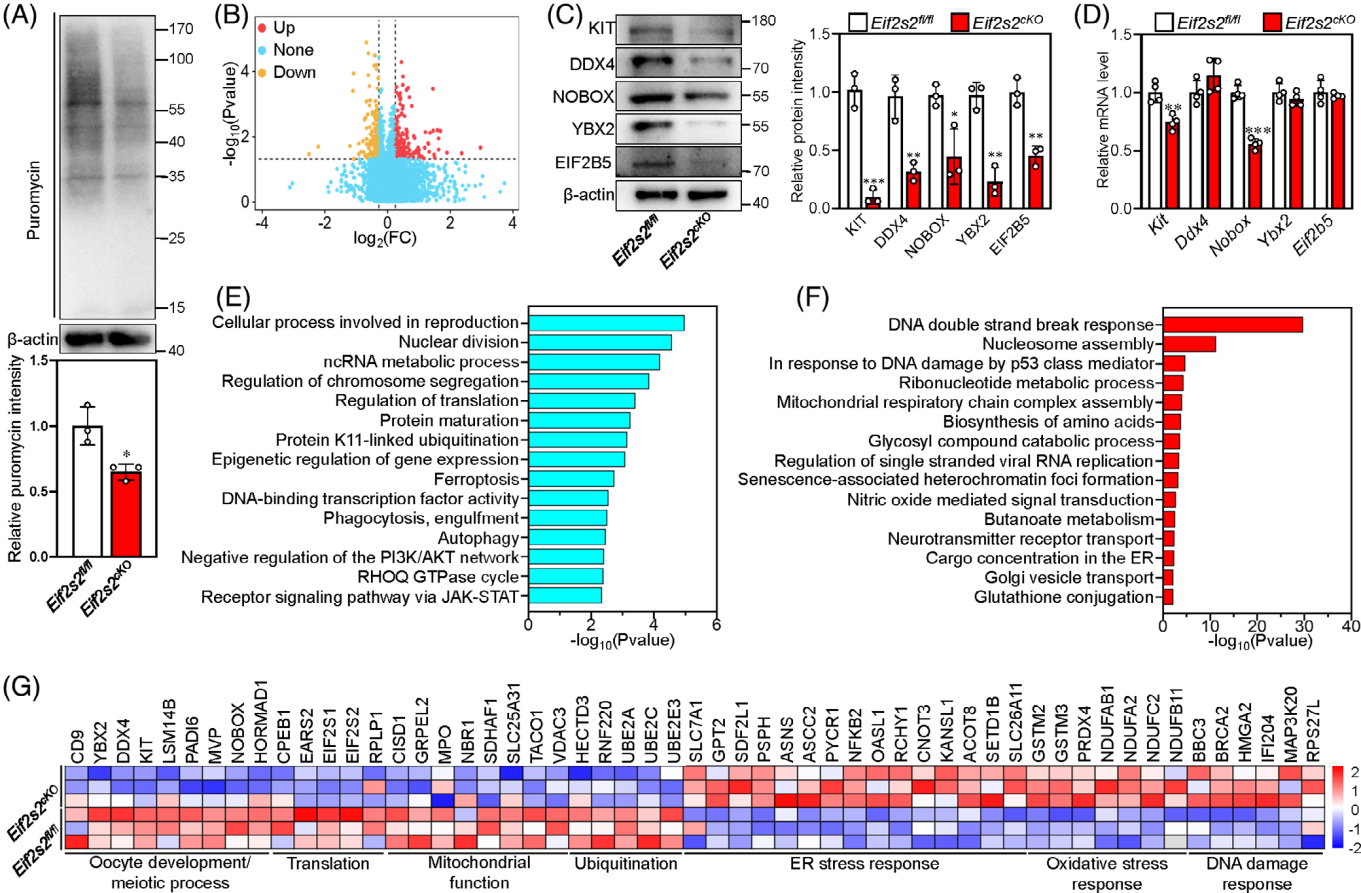
Deletion of *Eif2s2* results in the downregulation of proteins related to oocyte development and meiosis and the upregulation of stress response proteins. (A) The nascent protein synthesis level was detected in *Eif2s2* ^*fl/fl*^ and *Eif2s2* ^*cKO*^ ovaries at 1 dpp treated with 1 ng/mL puromycin for 1 h using an anti‐puromycin antibody. The intensity of nascent synthesized protein was quantified using β‐actin as an internal control. *n* = 3. (B) Volcano plot comparing the proteins of *Eif2s2* ^*fl/fl*^ and *Eif2s2* ^*cKO*^ ovaries at 1 dpp. Proteins that increased or decreased by >1.2‐fold in *Eif2s2* ^*cKO*^ ovaries are highlighted in red or orange, respectively. (C) Western blotting (WB) analysis of KIT, NOBOX, DDX4, YBX2, EIF2S1 and EIF2B5 expression within *Eif2s2* ^*fl/fl*^ and *Eif2s2* ^*cKO*^ mouse ovaries. β‐actin was utilized as an internal control. *n* = 3. (D) The mRNA levels of *Ddx4*, *Kit*, *Nobox*, *Ybx2*, *Eif2s1* and *Eif2b5* in ovaries from *Eif2s2* ^*fl/fl*^ and *Eif2s2* ^*cKO*^ mice at 1dpp. *n* = 4. (E and F) Bar graphs illustrating the enriched GO/KEGG terms or canonical pathways associated with the proteins that have decreased (E) or increased (F) levels in *Eif2s2* ^*cKO*^ ovaries. (G) Heatmaps depict differences between *Eif2s2* ^*fl/fl*^ and *Eif2s2* ^*cKO*^ ovaries in the expression of proteins involved in various processes. Bars represent the mean ± SD. A two‐sided Student's *t*‐test was performed to determine *p* values (*p < 0.05, **p < 0.01 and ***p < 0.001).

*Eif2s2* is crucial for translation initiation. We therefore detected the translational efficiency by the puromycin incorporation in 1‐dpp ovaries of *Eif2s2* ^*fl/fl*^ and *Eif2s2* ^*cKO*^ mice. The incorporation of puromycin into peptides/proteins decreased significantly in the ovaries of *Eif2s2* ^*cKO*^ mice compared to those of *Eif2s2* ^*fl/fl*^ mice (Figure 2A). Next, we analysed the protein‐expression profile in the ovaries by liquid chromatography–tandem mass spectrometry (LC–MS/MS). A total of 359 proteins (including 220 upregulated and 139 downregulated proteins) were differentially expressed in the ovaries of *Eif2s2* ^*cKO*^ mice compared with those of *Eif2s2* ^*fl/fl*^ mice (Figure 2B). Representative proteins that changed significantly were validated by WB analysis, such as KIT, DDX4, NOBOX, YBX2 and EIF2S1 (Figure 2C). The protein levels of EIF2S2 were significantly lower in the *Eif2s2* ^*cKO*^ ovaries than those in the *Eif2s2* ^*fl/fl*^ ovaries, consisting with our WB result (Figures 2G and S1C). The mRNA levels of DDX4, YBX2 and EIF2B5 validated (Figure 2D) showed no changes, indicating that the levels of these proteins are regulated at the translational level. The gene ontology (GO) analysis of the downregulated proteins revealed that the terms related to oocyte development or meiotic process, translation, mitochondrial function and ubiquitination were enriched (Figure 2E,G). In particular, the downregulation of NOBOX and KIT (Figure 2C,G) could lead to failure of primordial follicle formation and depletion of oocytes.^23^, ^24^ In addition, the downregulation of proteins involved in the ubiquitination indicated a downregulation of global ubiquitination level. In contrast, the upregulated proteins mainly participated in endoplasmic reticulum (ER) stress response, oxidative stress response and DNA damage response (Figure 2F,G). In particular, the upregulation of DNA damage response protein (BRCA2) and antioxidant proteins (GSTM2, GSTM3 and NDUFAB1) indicated enhanced DNA damage and ROS levels.^25^, ^26^ In summary, *Eif2s2* deletion in oocytes causes the insufficient synthesis of proteins related to oocyte growth and meiotic progression and upregulation of proteins related to ER stress, oxidative stress and DNA damage response.

### 2.3 Associated analysis between protein and gene expression

To determine whether *Eif2s2*‐deletion‐related proteomic changes were directly regulated by its role in translational instead of transcriptional regulation, we conducted the RNA‐seq analysis in *Eif2s2* ^*fl/fl*^ and *Eif2s2* ^*cKO*^ mouse ovaries at 1 dpp. In *Eif2s2* ^*cKO*^ mouse ovaries, it can be found that 240 genes were upregulated, and 243 genes were downregulated (Figure S2A). The representative genes were verified by qPCR (Figure S2B). Gene enrichment analysis revealed that downregulated genes in *Eif2s2* ^*cKO*^ mouse ovaries mainly controlled oogenic/meiotic process, and upregulated genes mainly participated in stress response, amino acid metabolism and glycolysis/oxidative phosphorylation (Figure S2C–F).

**FIGURE 3:**
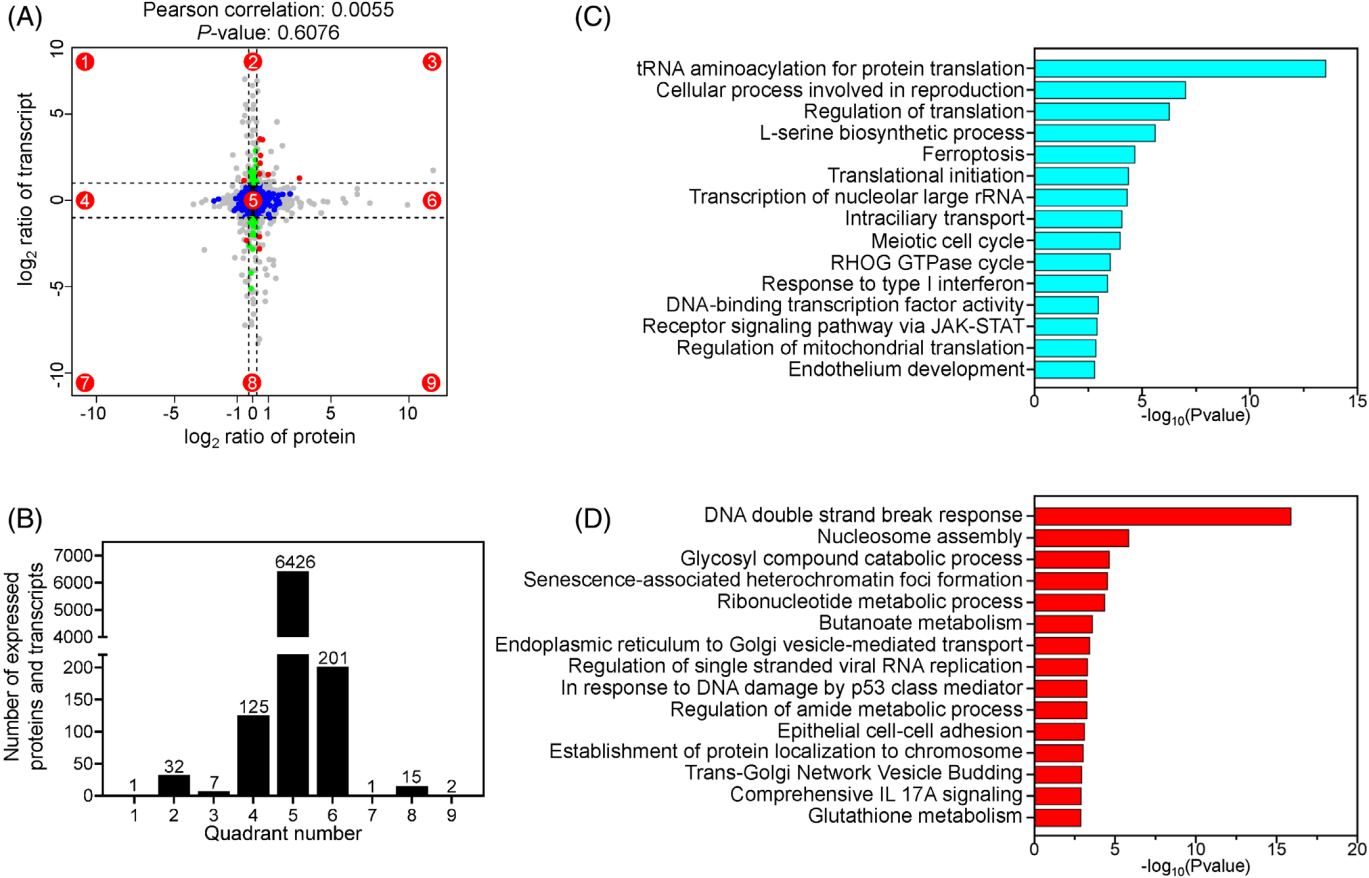
Combined transcriptomic and proteomic analysis in *Eif2s2* ^*fl/fl*^ and *Eif2s2* ^*cKO*^ ovaries. (A) The scatter plot illustrates the nine‐quadrant associative analysis of transcripts and proteins in *Eif2s2* ^*fl/fl*^ and *Eif2s2* ^*cKO*^ ovaries, where each dot represents a gene/protein. The dashed lines on the abscissa and ordinate represent the fold change (FC) thresholds for differentially expressed genes (DEGs; FC ≥2.0) and differentially abundant proteins (DAPs; FC ≥1.2), respectively. Genes/proteins outside these lines are considered to be DEGs/DAPs, whereas those within the lines are not. Quadrants are numbered 1–9 as indicated. Grey points indicate that the FC criterion was met without satisfying the p‐value criterion. The number of valid points in each quadrant showed in (B). (C, D) Bar graphs illustrating the enriched GO/KEGG terms or canonical pathways associated with the transcripts/proteins in quadrants 1, 2 and 4 (C) or in quadrants 6, 8 and 9 (D).

Then, we conducted a nine‐quadrant analysis by jointly analysing the proteome and transcriptome from *Eif2s2* ^*fl/fl*^ and *Eif2s2* ^*cKO*^ mouse ovaries. The diagram captured a total of 7011 proteins/genes (Figure 3A), and the number of proteins or transcripts per quadrant is presented in Figure 3B. In quadrant 5, 6426 proteins showed no significant difference in expression at the mRNA and protein levels. Quadrants 2 and 8 included 32 and 15 proteins, respectively, which were differentially expressed at the mRNA level only. In contrast, quadrants 4 and 6 featured 125 and 201 proteins, respectively, that were differentially expressed at the protein level only. Quadrants 1 and 9 included 1 and 9 proteins, respectively, which showed negative correlation between protein and mRNA levels. Quadrants 3 and 7 included 7 and 1 proteins, respectively, which were positive correlated. Pearson's correlation analysis revealed no correlation between the fold change (FC) in protein levels and the FC in transcript levels (rPearson = 0.0055, *p*‐value = 0.6076, Figure 3A). The proteins in quadrant 1, 2 and 4 were downregulated at the translational level, and the enrichment result showed other two terms related to the L‐serine biosynthetic process and intraciliary transport, which are related to mitochondrial function and microtubule‐based organelle transportation (Figure 3C). Proteins in quadrants 6, 8 and 9 were upregulated at the translational level, and the enrichment analysis revealed another term related to glutathione metabolism, a pathway activated in response to oxidative stress (Figure 3D).

### 2.4 Premeiotic *Eif2s2* deletion leads to oocyte meiotic arrest at the early diplotene stage

**FIGURE 4:**
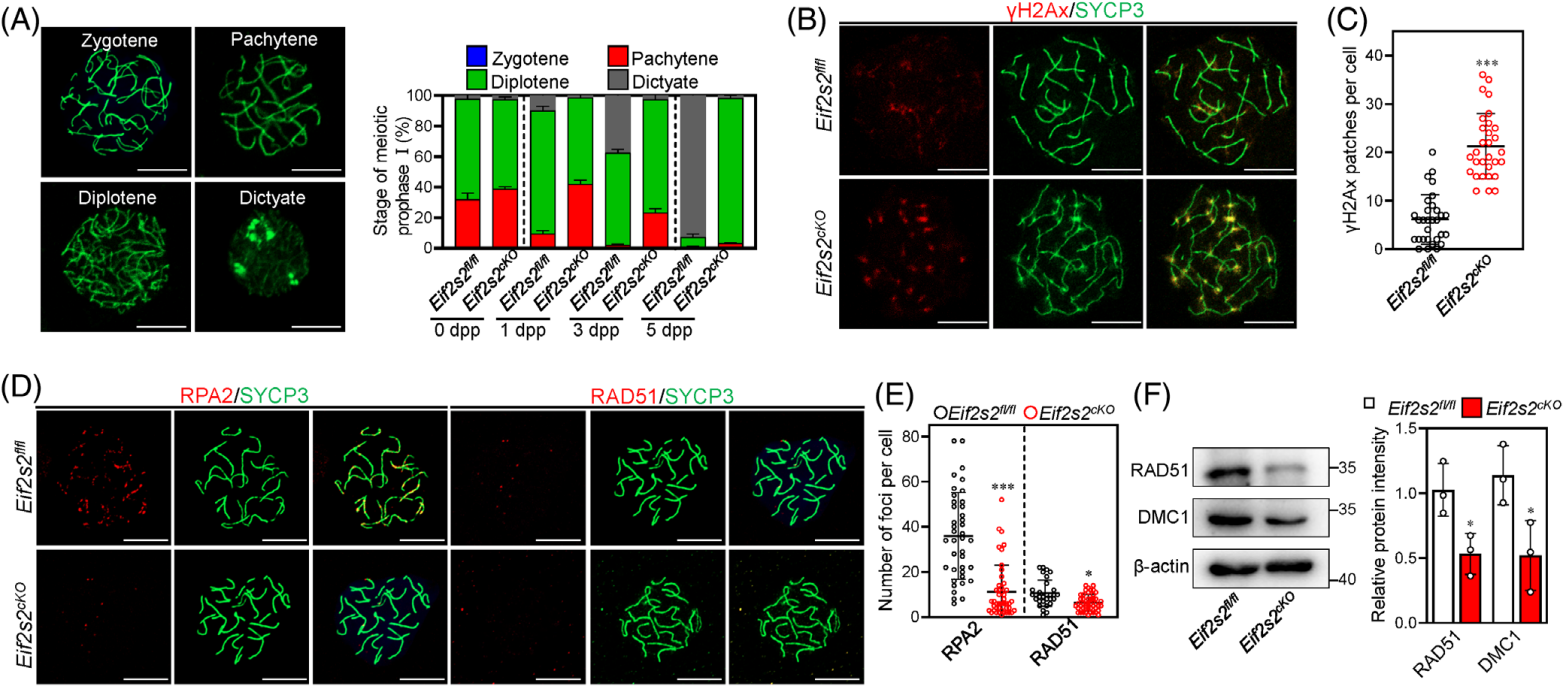
Depletion of *Eif2s2* in premeiotic germ cells cause failure of homologous recombination and oocytes arrest at the early diplotene stages. (A) Representative images of mouse oocyte nuclei at the zygotene, pachytene, diplotene and dictyate stages of meiosis prophase I. Surface spreads were stained with SYCP3 (green) immunolabelling. During the zygotene stage, chromosomes begin to align, initiating the formation of the synaptonemal complex; pachytene, paired homologous chromosomes undergo maximal shortening and thickening; diplotene, homologous chromosomes separate; dictyate, two to four nucleoli are clearly visible. The percentages of oocytes at various stages were shown at 0, 1, 3 and 5 days postpartum (dpp). 0 dpp: *Eif2s2* ^*fl/fl*^ *N* = 152, *Eif2s2* ^*cKO*^ *n* = 279. 1 dpp: *Eif2s2* ^*fl/fl*^ *N* = 396, *Eif2s2* ^*cKO*^ *n* = 327. 3 dpp: *Eif2s2* ^*fl/fl*^ *N* = 182, *Eif2s2* ^*cKO*^ *n* = 213. 5 dpp: *Eif2s2* ^*fl/fl*^ *N* = 156, *Eif2s2* ^*cKO*^ *n* = 198. (B, C) Immunofluorescence staining of oocyte nuclear chromosome spreads by anti‐γH2Ax (red, B) and anti‐SYCP3 (green, B) antibodies in *Eif2s2* ^*fl/fl*^ and *Eif2s2* ^*cKO*^ mouse ovaries at 0 dpp. Numbers of patches of γH2Ax in *Eif2s2* ^*fl/fl*^ (*n* = 30) and *Eif2s2* ^*cKO*^ (*n* = 30) mouse ovaries (C). (D, E) Immunofluorescence staining of oocyte nuclear chromosome spreads by anti‐RPA2 (red, left panel of D), anti‐RAD51 (red, right panel of D) and anti‐SYCP3 (green, D) antibodies in *Eif2s2* ^*fl/fl*^ and *Eif2s2* ^*cKO*^ mouse ovaries. Numbers of foci of RPA2 and RAD51 in *Eif2s2* ^*fl/fl*^ and *Eif2s2* ^*cKO*^ mouse ovaries (E). RPA2: *Eif2s2* ^*fl/fl*^ *N* = 45, *Eif2s2* ^*cKO*^ *n* = 45. RAD51: *Eif2s2* ^*fl/fl*^ *N* = 30, *Eif2s2* ^*cKO*^ *n* = 45. (F) Western blotting (WB) analysis of RAD51 and DMC1 expression in *Eif2s2* ^*fl/fl*^ and *Eif2s2* ^*cKO*^ mouse ovaries. β‐actin was utilized as an internal control. *n* = 3. Scale bar: 10 μM. Bars represent the mean ± SD. A two‐sided Student's *t*‐test was performed to determine *p* values (*p < 0.05, **p < 0.01 and ***p < 0.001).

The proteomic analysis and YBX2 immunostaining results showed that the proteins related to meiotic progression were downregulated. Therefore, we examined the meiosis prophase I progression by using surface spreading. The results showed an elevated percentage of pachytene oocytes in *Eif2s2* ^*cKO*^ mice compared to those in *Eif2s2* ^*fl/fl*^ mice at 1dpp (44.44% vs. 12.10%, Figure 4A). And, the percentage of dictyate stage oocytes in *Eif2s2* ^*cKO*^ mice was dramatically lower than those in *Eif2s2* ^*fl/fl*^ mice at 5 dpp (1.82% vs. 92.92%, Figure 4A). During meiosis prophase I, the homologous chromosomes synapse and form crossover through meiotic recombination. The surface spreading staining results showed that the number of γH2Ax patches were significantly increased in the nuclei of oocytes from *Eif2s2* ^*cKO*^ mice compared to those from *Eif2s2* ^*fl/fl*^ mice at 0 dpp, indicating an increased number of unrepaired DSBs (Figure 4B,C). Moreover, the foci numbers of RPA2 and RAD51, and the protein levels of DMC1 and RAD51 were significantly decreased (Figure 4D–F), but the percentage of unsynapsed oocytes was not different between *Eif2s2* ^*cKO*^ and *Eif2s2* ^*fl/fl*^ mice (Figure S3A,B). These results suggest that HR deficiency caused oocyte pachytene arrest at 1 dpp in *Eif2s2* ^*cKO*^ mice, which may further lead to meiotic arrest at the early diplotene stage at 5 dpp.

### 2.5 *Eif2s2* deletion induces integrated stress responses in oocytes

**FIGURE 5:**
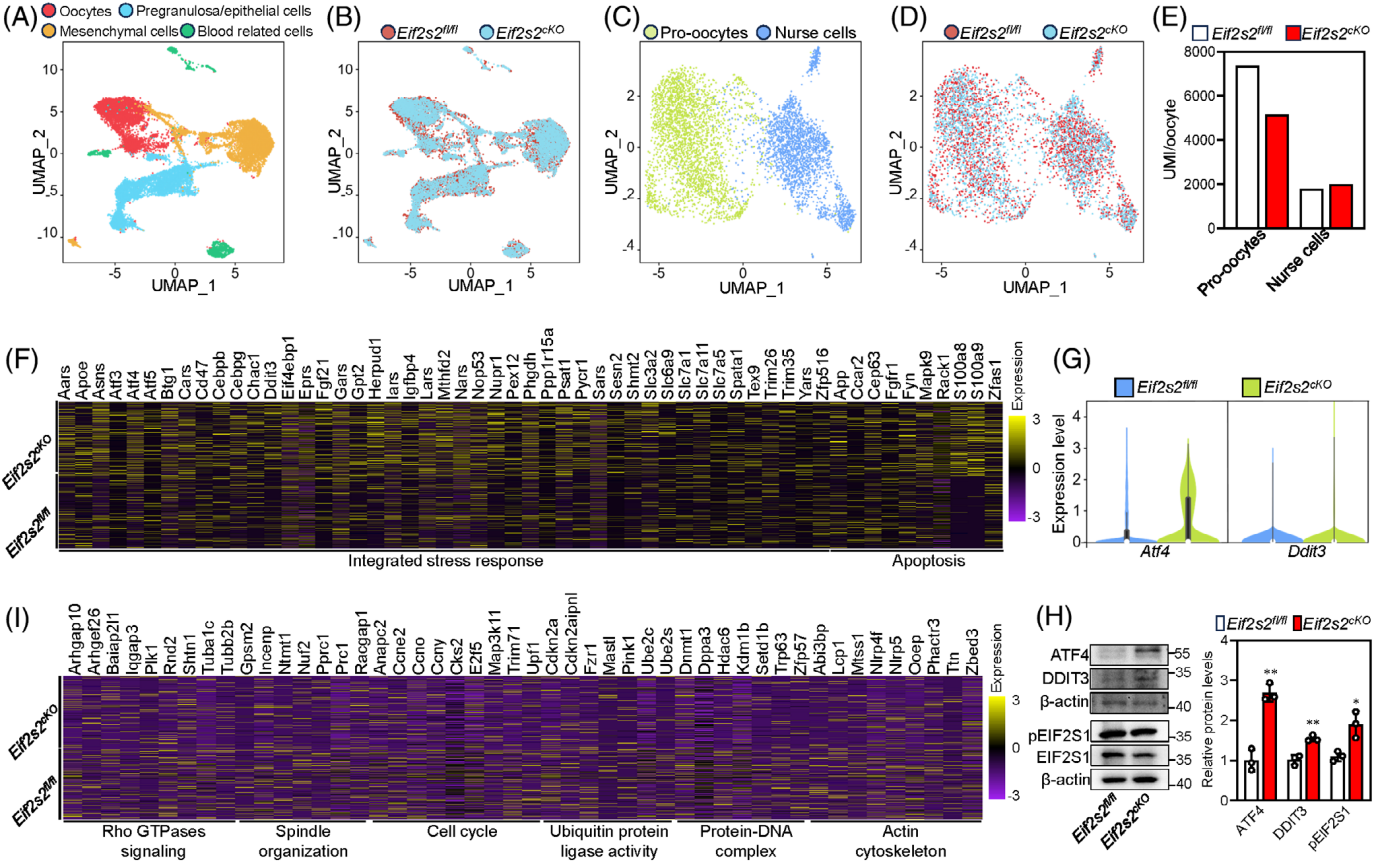
scRNA‐seq analysis reveals the activation of ISR signalling pathway in ovaries of *Eif2s2* ^*cKO*^ mice. (A, B) uMAP plots of ovarian cells based on four cell types (germ cells, pregranulosa cells, mesenchymal cells and blood‐related cells; (A) and sample groups (B). (C, D) Clustering of germ cell population with uMAP, coloured based on cell types (pro‐oocytes and nurse cells, C) and sample groups (D). (E) Total nUMI/cell for pro‐oocytes and nurse cells in *Eif2s2* ^*fl/fl*^ and *Eif2s2* ^*cKO*^ ovaries. (F) The relative expression of the genes upregulated in the pro‐oocyte clusters of *Eif2s2* ^*cKO*^ mouse ovaries. (G) Violin plots of *Atf4* and *Ddit3* expression in *Eif2s2* ^*fl/fl*^ and *Eif2s2* ^*cKO*^ pro‐oocytes at 1 dpp. (H) Western blotting (WB) analysis of ATF4, DDIT3, pEIF2S1 and EIF2S1 expression within *Eif2s2* ^*fl/fl*^ and *Eif2s2* ^*cKO*^ mouse ovaries. β‐actin was utilized as an internal control. *n* = 3. (I) The relative expression of the genes downregulated in pro‐oocytes clusters of *Eif2s2* ^*cKO*^ mouse ovaries. Bars represent the mean ± SD. A two‐sided Student's *t*‐test was performed to determine *p* values (*p < 0.05, **p < 0.01 and ***p < 0.001).

To investigate the role of EIF2S2 in primordial follicle formation, we performed single‐cell RNA‐sequencing (scRNA‐seq) on 1‐dpp *Eif2s2* ^*fl/fl*^ and *Eif2s2* ^*cKO*^ mouse ovaries. Following the preparation and barcoding of ovarian cell populations after single‐cell capture, RNA sequencing was conducted. After the exclusion of low‐quality cells, the remaining number of cells were 8762 in the *Eif2s2* ^*fl/fl*^ mouse and 8695 in the *Eif2s2* ^*cKO*^ mouse. Nineteen cell clusters were generated using the UMAP technology (Figure S4A). According to the distinct transcriptomic signatures (Figure S4B,C), these cells could be subdivided as follows: oocytes, pregranulosa/epithelial cells, mesenchymal cells, blood related cells (Figure 5A). A high concordance of the uMAP plots (Figure 5B) and similar proportions, as well as cell numbers, of the four cell clusters (Figure S4D) were observed in *Eif2s2* ^*fl/fl*^ and *Eif2s2* ^*cKO*^ mouse ovaries. In‐depth analysis of the oocyte clusters revealed seven subclusters, classified as pro‐oocytes and nurse cells (Figures 5C and S4E), based on the nUMI (Figure S4F) and well‐characterized meiotic‐stage‐enriched transcripts (Figure S4G). A high concordance of the uMAP plots (Figure 5D) and the similar proportions of pro‐oocytes (Figure S4H–J) were observed in *Eif2s2* ^*fl/fl*^ and *Eif2s2* ^*cKO*^ mouse oocytes. The levels of UMI/cell were similar in nurse cells, but were obviously decreased in pro‐oocytes of *Eif2s2* ^*cKO*^ mice (Figure 5E). Differential gene expression analysis showed 1133 altered transcripts in *Eif2s2* ^*cKO*^ pro‐oocytes (727 downregulated and 406 upregulated, Figure S5A). Representative genes were validated in Figure S2B. The upregulated genes in *Eif2s2* ^*cKO*^ pro‐oocytes mainly participated in the integrated stress response (ISR) and apoptosis process (Figures 5F and S5B), with elevated levels of *Atf4* and *Ddit3* mRNA and phosphorylated EIF2S1, ATF4 and DDIT3 proteins (Figure 5G,H). The downregulated genes in *Eif2s2* ^*cKO*^ pro‐oocytes mainly participated in Rho GTPases signalling, cytoskeleton assembly and cell cycle phase transition (Figures 5I and S5C). Downregulation of these genes in crucial processes might impede the transportation of organelle and the primordial follicle formation.^5^

### 2.6 The depletion of *Eif2s2* decreases the expression of dictyate genes and impairs the communication between oocytes and pregranulosa cells

**FIGURE 6:**
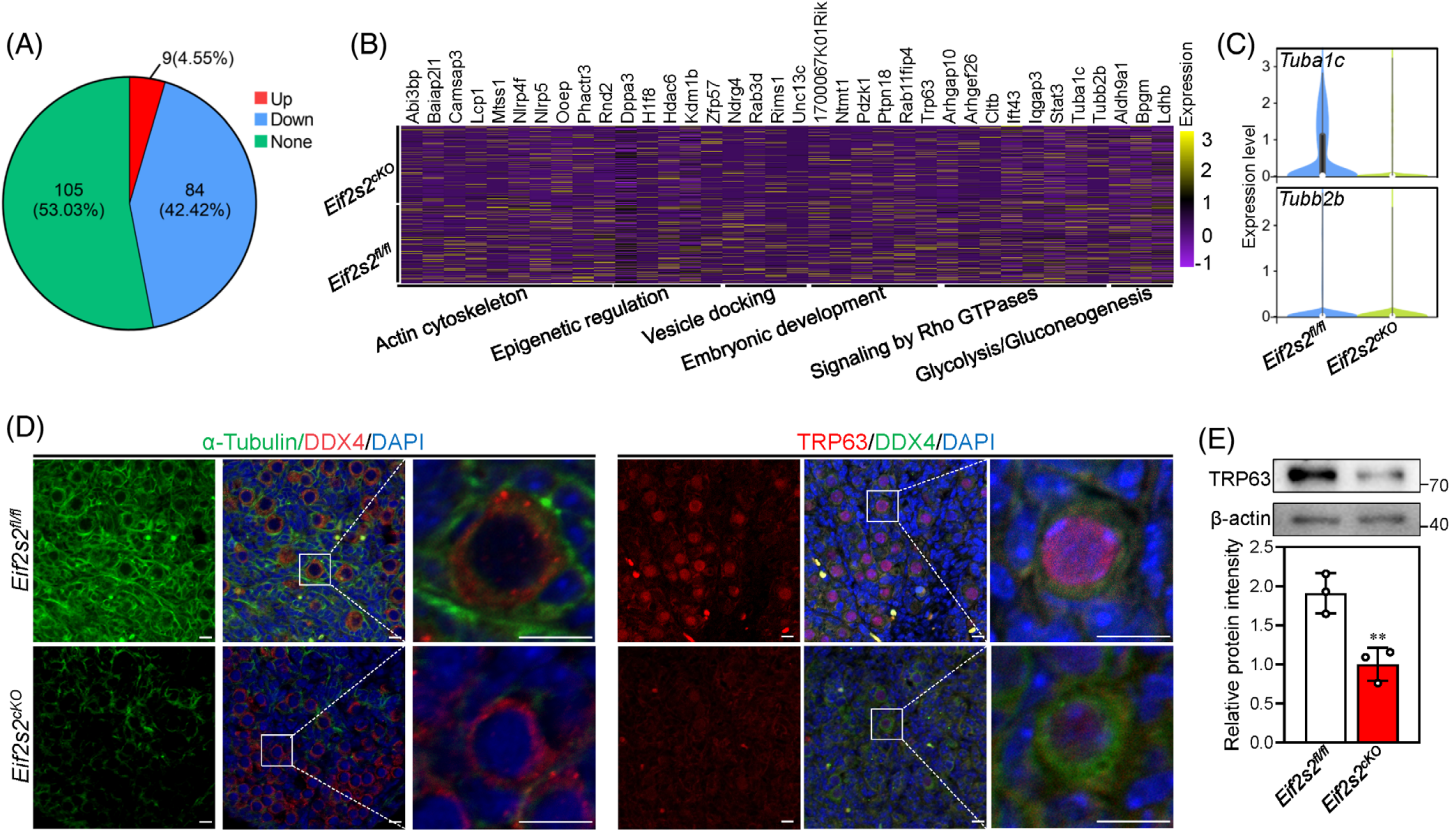
Premeiotic *Eif2s2* deletion in oocytes causes downregulation of dictyate genes. (A) Pie chart showing the number and percentage of upregulated, downregulated and unchanged dictyate genes in *Eif2s2* ^*cKO*^ mouse pro‐oocytes. (B) The relative expression of representative downregulated dictyate genes in pro‐oocyte clusters of *Eif2s2* ^*cKO*^ mouse ovaries. (C) Violin plots of *Tuba1c* and *tubb2b* expression in *Eif2s2* ^*fl/fl*^ and *Eif2s2* ^*cKO*^ pro‐oocytes at 1 dpp. (D) Immunostaining of α‐tubulin (green) and DDX4 (red), and TRP63 (red) and DDX4 (green) in *Eif2s2* ^*fl/fl*^ and *Eif2s2* ^*cKO*^ mouse ovaries. DAPI, blue. (E) Western blotting (WB) analysis of TRP63 expression within *Eif2s2* ^*fl/fl*^ and *Eif2s2* ^*cKO*^ mouse ovaries. β‐actin was utilized as an internal control. *n* = 3. Scale bars: 10 μm. Bars represent the mean ± SD. A two‐sided Student's *t*‐test was performed to determine *p* values (*p < 0.05, **p < 0.01 and ***p < 0.001).

We compared the differentially expressed transcripts related to dictyate stage in *Eif2s2* ^*fl/fl*^ and *Eif2s2* ^*cKO*^ pro‐oocytes. Among 198 dictyate genes,^5^ 84 genes were downregulated and nine genes were upregulated in *Eif2s2* ^*cKO*^ pro‐oocytes (Figure 6A). The downregulation of nearly 42% dictyate genes is consistent to the meiotic arrest of oocytes at the early diplotene stage (Figures 6A and 4A). In addition, the downregulation of tubulin (*Tuba1c* and *Tubb2b*) and *Trp63* genes corresponded with the decreases of alpha‐tubulin intensities and TRP63 levels in *Eif2s2* ^*cKO*^ oocytes, respectively (Figure 6B–E). In oocytes of *Eif2s2* ^*cKO*^ mice, the decreased percentage of oocytes with compacted Balbiani body may be due to the downregulation of tubulin levels.^5^

**FIGURE 7:**
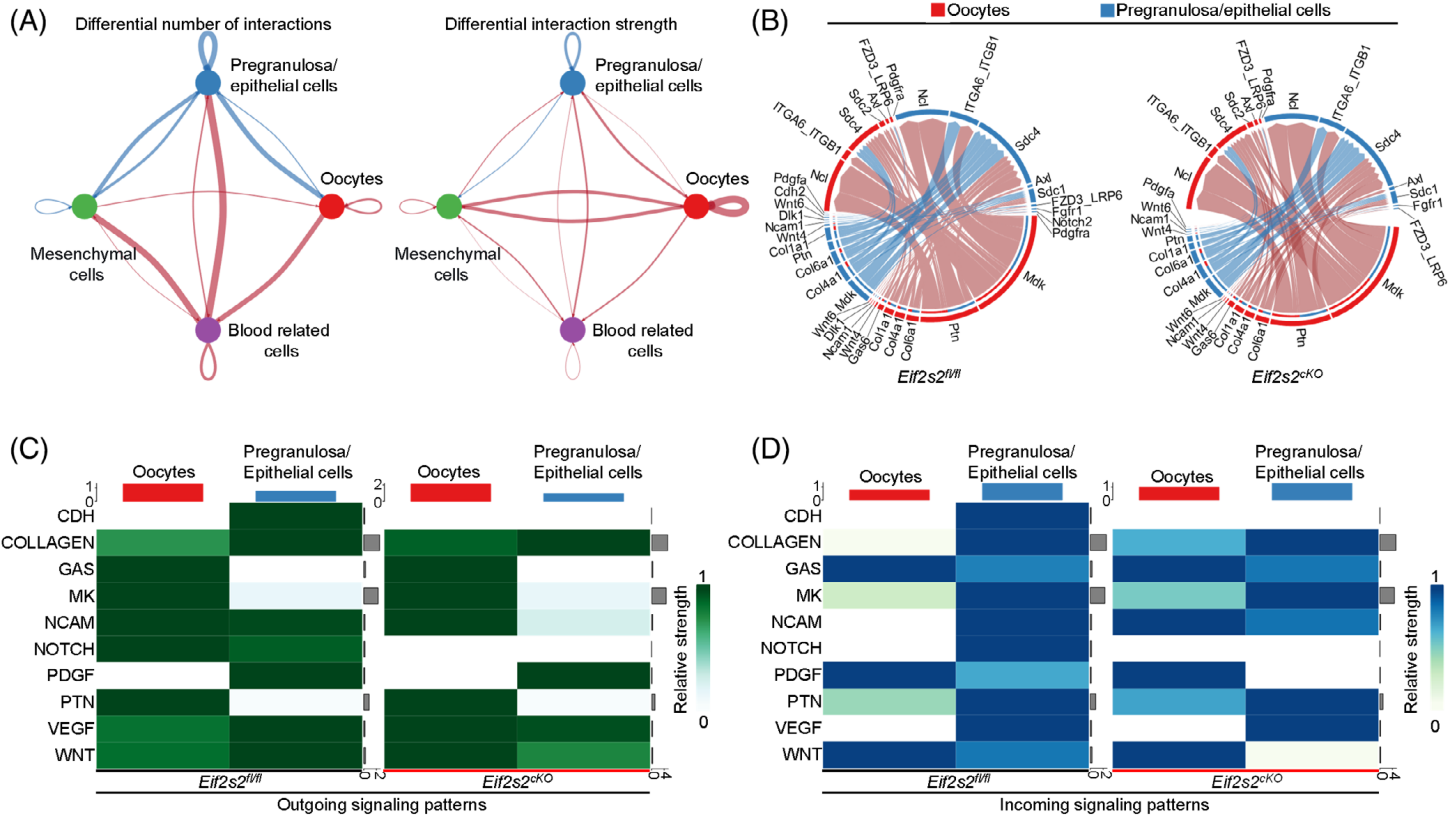
Premeiotic *Eif2s2* deletion in oocytes impairs communication between oocytes and pregranulosa/epithelial cells. (A) Differential interaction number and strength among the four ovarian cell clusters in ovaries from *Eif2s2* ^*fl/fl*^ and *Eif2s2* ^*cKO*^ mice. The red and blue lines indicate increase or decrease of number or strength, respectively, in ovaries of *Eif2s2* ^*cKO*^ mice compared to those in *Eif2s2* ^*fl/fl*^ mice. (B) Circular plots demonstrating the communication probabilities of representative differentially changed ligand–receptor pairs between oocytes and pregranulosa/epithelial cells in *Eif2s2* ^*fl/fl*^ and *Eif2s2* ^*cKO*^ ovaries. (C and D) Heat maps showed outgoing (C) and incoming (D) communication patterns of oocytes and pregranulosa/epithelial cells clusters of *Eif2s2* ^*fl/fl*^ and *Eif2s2* ^*cKO*^ ovaries.

During primordial follicle formation, oocytes and somatic cells frequently communicate with each other. Therefore, we analysed the cell‐to‐cell communication in *Eif2s2* ^*fl/fl*^ and *Eif2s2* ^*cKO*^ mouse ovaries using cell‐chat. Compared to those in *Eif2s2* ^*fl/fl*^ mice, premeiotic *Eif2s2* deletion altered the signalling patterns among ovarian cells, notably reducing the interactions between oocytes and pregranulosa/epithelial cells, as well as between pregranulosa/epithelial cells themselves (Figure 7A). Then, we identified altered ligand–receptor pairs between oocytes and pregranulosa/epithelial cells, and found that 89 ligand–receptor pairs were altered significantly in *Eif2s2* ^*cKO*^ mice compared to those in *Eif2s2* ^*fl/fl*^ mice (Figures 7B and S6A). In particular, the outgoing and/or incoming signalling strength of the Notch pathway in oocytes, as well as the Notch, WNT and CDH pathways in pregranulosa/epithelial cells, was decreased in *Eif2s2* ^*cKO*^ mice compared to that in *Eif2s2* ^*fl/fl*^ mice (Figure 7C,D), indicating that these pathways were inhibited in the ovaries of *Eif2s2* ^*cKO*^ mice. The inhibition of Notch, WNT and CDH pathways in the ovary could lead to failure of primordial follicle formation.^2^, ^27^

### 2.7 *Eif2s2* deletion causes dysfunction of mitochondria

**FIGURE 8:**
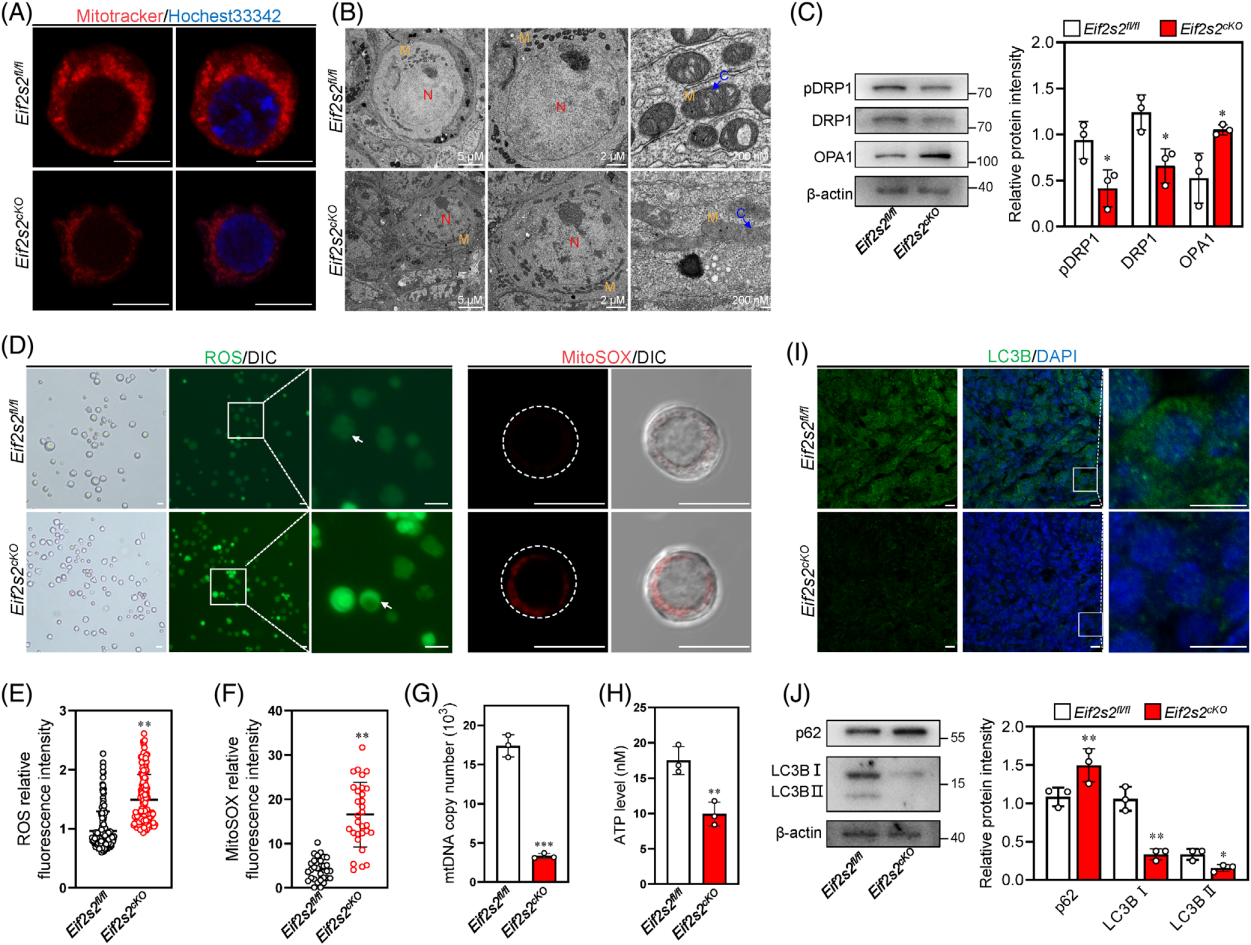
*Eif2s2* deletion in oocytes causes mitochondrial dysfunction. (A) The representative images for the Mitotracker intensity in oocytes from *Eif2s2* ^*fl/fl*^ and *Eif2s2* ^*cKO*^ mice. (B) Transmission electron microscopy (TEM) images of primordial follicles, nucleus and mitochondria from *Eif2s2* ^*fl/fl*^ and *Eif2s2* ^*cKO*^ mouse at 3 dpp. C, cristae of mitochondria; M, mitochondria; N, nucleus. Scale bars were indicated in the graphs. (C) Western blotting (WB) analysis of DRP1, pDRP1 and OPA1 expression within *Eif2s2* ^*fl/fl*^ and *Eif2s2* ^*cKO*^ mouse ovaries. β‐actin was utilized as an internal control. (D–F) The fluorescence staining of ROS using DCFH‐DA (green, D) and mitochondrial superoxide using MitoSOX (red, D), and relative fluorescence intensity of ROS (E) and MitoSOX (F) in oocytes of *Eif2s2* ^*fl/fl*^ and *Eif2s2* ^*cKO*^ mice. ROS: *Eif2s2* ^*fl/fl*^ *n* = 151, *Eif2s2* ^*cKO*^ *n* = 174. MitoSOX: *Eif2s2* ^*fl/fl*^ *n* = 31, *Eif2s2* ^*cKO*^ *n* = 30. (G and H) The mtDNA copy numbers (G) and the ATP levels (H) in oocytes of *Eif2s2* ^*fl/fl*^ and *Eif2s2* ^*cKO*^ mice. *n* = 3. (I) Immunostaining of LC3B (green) in *Eif2s2* ^*fl/fl*^ and *Eif2s2* ^*cKO*^ mouse ovaries. DAPI, blue. (J) WB analysis of p62 and LC3B expression within *Eif2s2* ^*fl/fl*^ and *Eif2s2* ^*cKO*^ mouse ovaries. β‐actin was utilized as an internal control. *n* = 3. Scale bar: 10 μm (A, D, I). Bars represent the mean ± SD. A two‐sided Student's *t*‐test was performed to determine *p* values (*p < 0.05, **p < 0.01 and ***p < 0.001).

The proteomic results showed a decrease of proteins related to mitochondrial function. Therefore, we detected the distribution of mitochondria using Mitotracker, and the results showed that less clustered mitochondria were observed in the cytoplasm of oocytes of *Eif2s2* ^*cKO*^ mice compared to those of *Eif2s2* ^*fl/fl*^ mice (Figure 8A). Furthermore, subcellular structures of oocytes were investigated by transmission electron microscope (TEM). In the ovaries of *Eif2s2* ^*fl/fl*^ mice, the oocytes contained healthy mitochondria with clear cristae and a Balbiani body (Figure 8B). However, in the ovaries of *Eif2s2* ^*cKO*^ mice, the oocytes contained elongated mitochondria with few cristae and no Balbiani body formation (Figure 8B). Consistent with this, the levels of DRP1 and phosphorylated DRP1 were significantly decreased, and the levels of OPA1 were significantly increased (Figure 8C). These results indicate that the elongation of mitochondria is due to the imbalance of mitochondrial fission/fusion process. Both the reactive oxygen species (ROS) and mitochondrial superoxide levels were significantly increased in oocytes from *Eif2s2* ^*cKO*^ mice compared to those from *Eif2s2* ^*fl/fl*^ mice (Figure 8D–F). Meanwhile, the copy number of mitochondrial DNA and ATP levels were significantly decreased in oocytes from *Eif2s2* ^*cKO*^ mice compared to those from *Eif2s2* ^*fl/fl*^ mice (Figure 8G,H). The clearance of damaged mitochondria is related to autophagic activities. The levels of LC3B‐II and the number of LC3B puncta were significantly reduced, and the levels of p62 were significantly raised in ovaries of *Eif2s2* ^*cKO*^ mice compared to those of *Eif2s2* ^*fl/fl*^ mice (Figure 8I,J), indicating the downregulation of autophagic activities in oocytes from *Eif2s2* ^*cKO*^ mice. In conclusion, *Eif2s2* deletion causes the dysfunction of mitochondria in oocytes.

### 2.8 *Eif2s2* deletion increases DNA damage and activates p53‐PUMA‐BAX apoptosis pathway in oocytes

**FIGURE 9:**
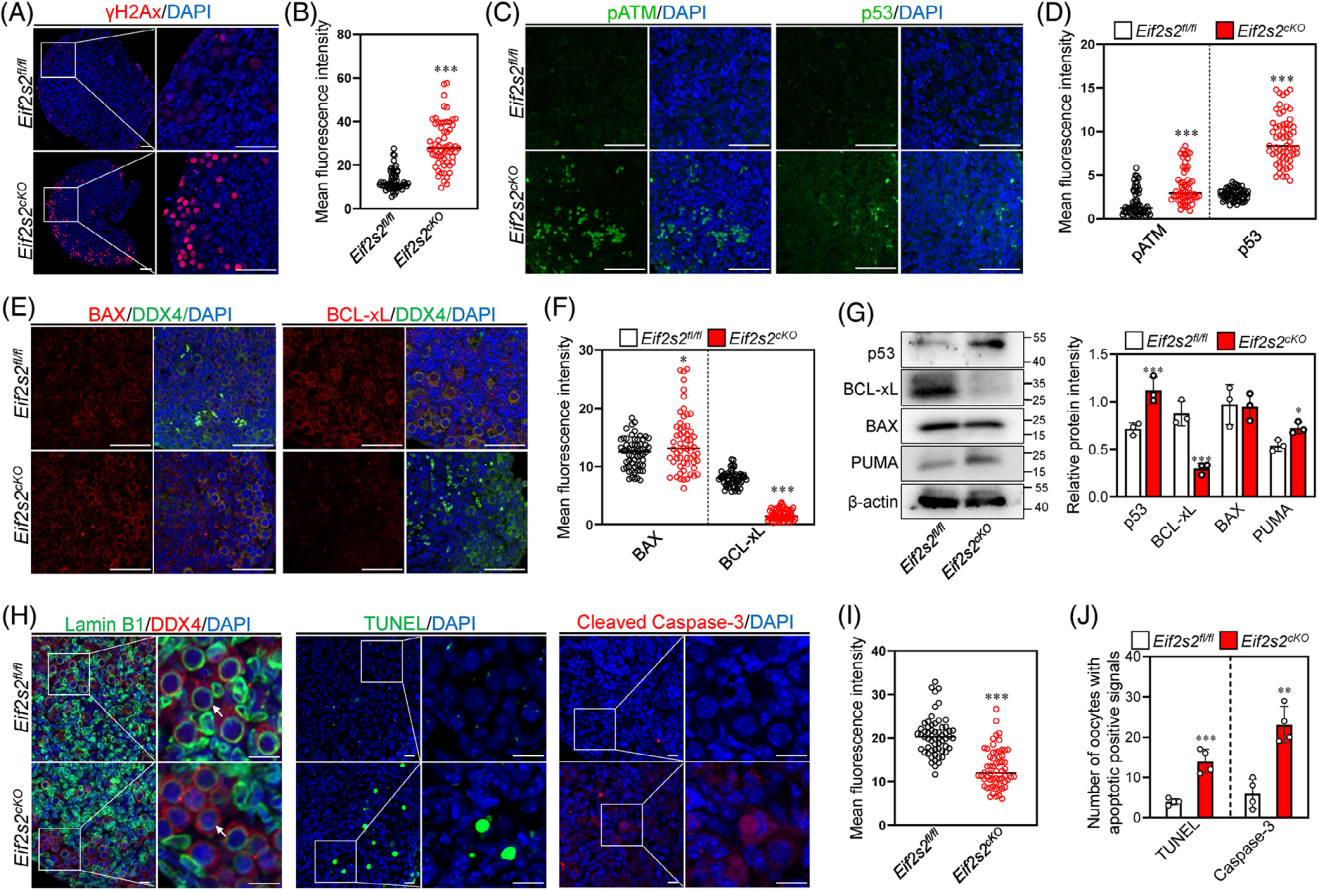
*Eif2s2* deletion in oocytes causes DNA damage and activate the p53‐PUMA‐BAX apoptotic pathway. (A and B) Immunostaining of γH2Ax (red) in *Eif2s2* ^*fl/fl*^ and *Eif2s2* ^*cKO*^ mouse ovaries (A). DAPI, blue. Scale bar: 50 μM. Mean fluorescent intensity of γH2Ax in oocytes (B). *Eif2s2* ^*fl/fl*^ *n* = 60, *Eif2s2* ^*cKO*^ *n* = 60. (C and D) Immunostaining of pATM (green, left panel of C) and p53 (green, right panel of C). DAPI, blue. Scale bar: 50 μM. Mean fluorescent intensity of pATM and p53 in oocytes (D). *Eif2s2* ^*fl/fl*^ *n* = 60, *Eif2s2* ^*cKO*^ *n* = 60. (E and F) Immunostaining of BAX (red, left panel of E) and BCL‐xL (red, right panel of E). DAPI, blue. Scale bar: 50 μM. Mean fluorescent intensity of BAX and BCL‐xL in oocytes (F). *Eif2s2* ^*fl/fl*^ *n* = 60, *Eif2s2* ^*cKO*^ *n* = 60. (G) Western blotting (WB) analysis of p53, RAD51, BCL‐xL, BAX and PUMA expression within *Eif2s2* ^*fl/fl*^ and *Eif2s2* ^*cKO*^ mouse ovaries. β‐actin was used as an internal control. *n* = 3. (H, I, J) Immunostaining of Lamin B1 (green, H), TUNEL (green, H), cleaved Caspase‐3 (red, H) and DDX4 (red, H) in *Eif2s2* ^*fl/fl*^ and *Eif2s2* ^*cKO*^ mouse ovaries. DAPI, blue. Scale bar: 10 μM. Mean fluorescent intensity of Lamin B1 in oocytes (I). *Eif2s2* ^*fl/fl*^ *n* = 60, *Eif2s2* ^*cKO*^ *n* = 60. The number of oocytes exhibiting TUNEL‐ or cleaved Caspase‐3‐positive signals in the ovaries of *Eif2s2* ^*fl/fl*^ and *Eif2s2* ^*cKO*^ mouse were shown in (J). *n* = 4. Bars represent the mean ± SD. A two‐sided Student's *t*‐test was performed to determine *p* values (*p < 0.05, **p < 0.01 and ***p < 0.001).

Excessive ROS accumulation can induce DNA damages. Therefore, we determined the DNA damage levels in the ovary by γH2Ax immunostaining. The whole‐mount staining results showed that the intensities of γH2Ax were significantly increased in the nuclei of oocytes from *Eif2s2* ^*cKO*^ mice compared to those from *Eif2s2* ^*fl/fl*^ mice, indicating an increased level of DNA damage at 1dpp (Figure 9A,B). Next, we detected the expression of apoptosis‐related proteins. The phosphorylated ATM intensities and p53 levels were significantly increased, BCL‐xL intensities and levels were significantly decreased and PUMA levels and BAX intensities were increased in the ovaries of *Eif2s2* ^*cKO*^ mice compared to those of *Eif2s2* ^*fl/fl*^ mice (Figure 9C–G). Thus, the *Eif2s2* deletion activates the p53‐PUMA‐BAX apoptotic pathway. Consistent with this, the intensity of Lamin B1 in oocytes was significantly decreased, and both oocytes and cells with positive cleaved‐Caspase‐3 and TUNEL signals were increased in the *Eif2s2* ^*cKO*^ mice compared to those in *Eif2s2* ^*fl/fl*^ mice (Figures 9H–J and S7A,B), suggesting the occurrence of oocyte apoptosis in *Eif2s2* ^*cKO*^ mice.

### 2.9 Stress‐induced integrated stress response leads to reduced number of oocytes and primordial follicles in the ovaries of newborn mice

**FIGURE 10:**
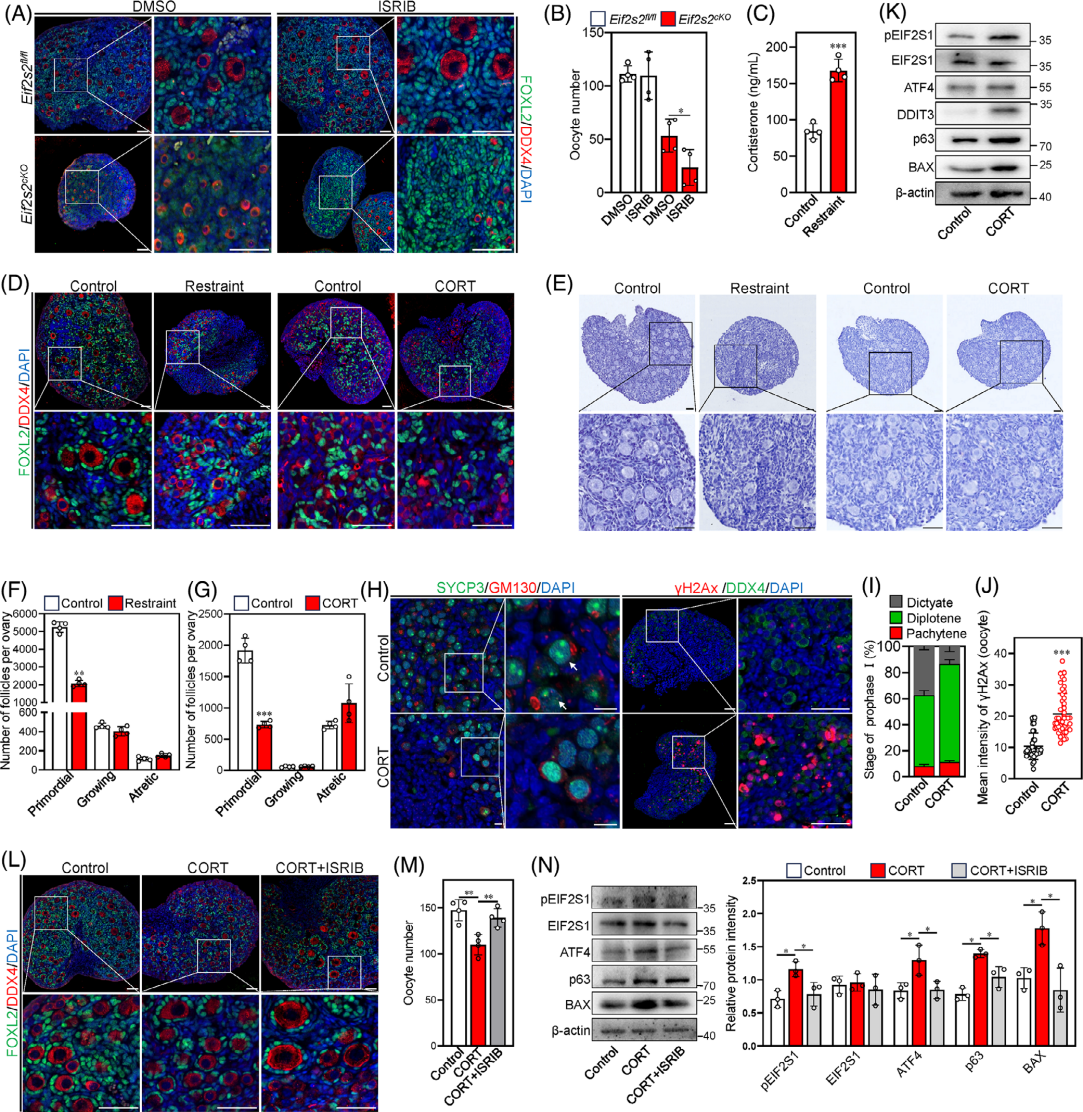
Stress‐induced corticosterone causes apoptosis of oocytes and primordial follicles in newborn mouse ovaries. (A and B) Ovaries from *Eif2s2* ^*fl/fl*^ and *Eif2s2* ^*cKO*^ mice at 1dpp were cultured in medium supplemented with DMSO or ISRIB for 3 days, respectively. Morphological comparison of the ovaries (A) and the number of oocytes (B) in *Eif2s2* ^*fl/fl*^ and *Eif2s2* ^*cKO*^ mice. FOXL2, green; DDX4, red; DAPI, blue. *n* = 4. (C) Serum corticosterone (CORT) levels of control and restraint group mice. *n* = 4. (D) Morphological comparison of ovaries of control (left panel) and restraint group (left panel) mice, and ovaries of control (right panel) and CORT injection group (CORT, right panel). DDX4, red; FOXL2, green; DAPI, blue. Scale bars: 50 μm. (E, F and G) Morphological comparison of ovaries (E) and the number of primordial, growing and atretic follicles in the control and restraint group (F), and in the control and CORT group (G). Nuclei were stained by haematoxylin. *n* = 4. Scale bars: 50 μm. (H) Representative images of SYCP3 (green) and GM130 (red) double staining and images of γH2Ax (red) and DDX4 (green) double staining. DAPI, blue. Scale bar: 50 μM. The arrow indicates SYCP3 cluster in dictyate oocytes. (I) The statistical results of the percentage of oocytes at different meiosis prophase I stages in the control and CORT groups at 48 h after CORT injection. *n* = 3. (J) Relative intensity of γH2Ax in control and CORT group mouse oocytes at 48 h after CORT injection. Control, *n* = 45; CORT, *n* = 45. (K) Western blotting (WB) analysis of pEIF2S1, EIF2S1, ATF4, DDIT3, p63 and BAX expression in control and CORT group mouse ovaries at 48 h after CORT injection. β‐actin was utilized as an internal control. *n* = 3. (L and M) Mice were injected with ethanol (control), 30 mg/kg corticosterone (CORT) and 30 mg/kg corticosterone +1 μM ISRIB (CORT+ISRIB). (L) shows a morphological comparison of the ovaries, and (M) details the number of oocytes in each group. FOXL2, green; DDX4, red; DAPI, blue. (N) WB analysis of pEIF2S1, EIF2S1, ATF4, p63 and BAX expression within control, CORT and CORT+ISRIB group mouse ovaries. β‐actin was utilized as an internal control. *n* = 3. Bars represent the mean ± SD. A two‐sided Student's *t*‐test was performed to determine *p* values (*p < 0.05, **p < 0.01 and ***p < 0.001).

The scRNA‐seq and WB results indicate the occurrence of ISR in oocytes of *Eif2s2* ^*cKO*^ mice. Therefore, we treated the ovaries of mice at 1 dpp with ISR inhibitor (ISRIB) to find out the effect of ISR on the survival of oocytes from *Eif2s2* ^*cKO*^ mice. However, a more severe oocyte loss was observed in the ovaries of *Eif2s2* ^*cKO*^ mice treated with ISRIB compared to those of *Eif2s2* ^*cKO*^ mice treated with DMSO (Figure 10A,B), possibly due to the persistent ISR induced by *Eif2s2*‐deletion. Next, we constructed a maternal restraint stress model to further investigate the impact of ISR on primordial follicle formation in newborn mice. Compared to those in the control group, the stress significantly decreased the number of oocytes and primordial follicles in newborn mice at 4 dpp, accompanied by significantly increased levels of the stress hormone corticosterone (CORT) in serum (Figures 10C–F and S8A). Next, we injected newborn mice with 30 mg/kg CORT to induce stress. Compared to those of the control group, the percentage of dictyate oocytes and the number of primordial follicles were significantly decreased, and the intensities of γH2Ax in oocytes and the levels of p63, BAX, pEIF2S1, ATF4 and DDIT3 were significantly increased in the CORT group (Figures 10D,E,G–K and S8B,C). However, treatment with 10 or 50 μM CORT in vitro did not affect the number of primordial, growing and atretic follicles (Figure S8D,E), suggesting that CORT impairs the formation of primordial follicles indirectly. Furthermore, ISRIB could reverse the loss of oocyte induced by CORT (Figure 10L,M). Consistent with this, ISRIB reversed the upregulation of protein levels of pEIF2S1, ATF4, DDIT3, p63, BAX induced by CORT treatment (Figure 10N). These results suggest that stress induces the apoptosis of oocytes and primordial follicles in newborn mouse ovaries possibly by the elevation of serum CORT levels and activating ISR.

## 3 DISCUSSION

**FIGURE 11:**
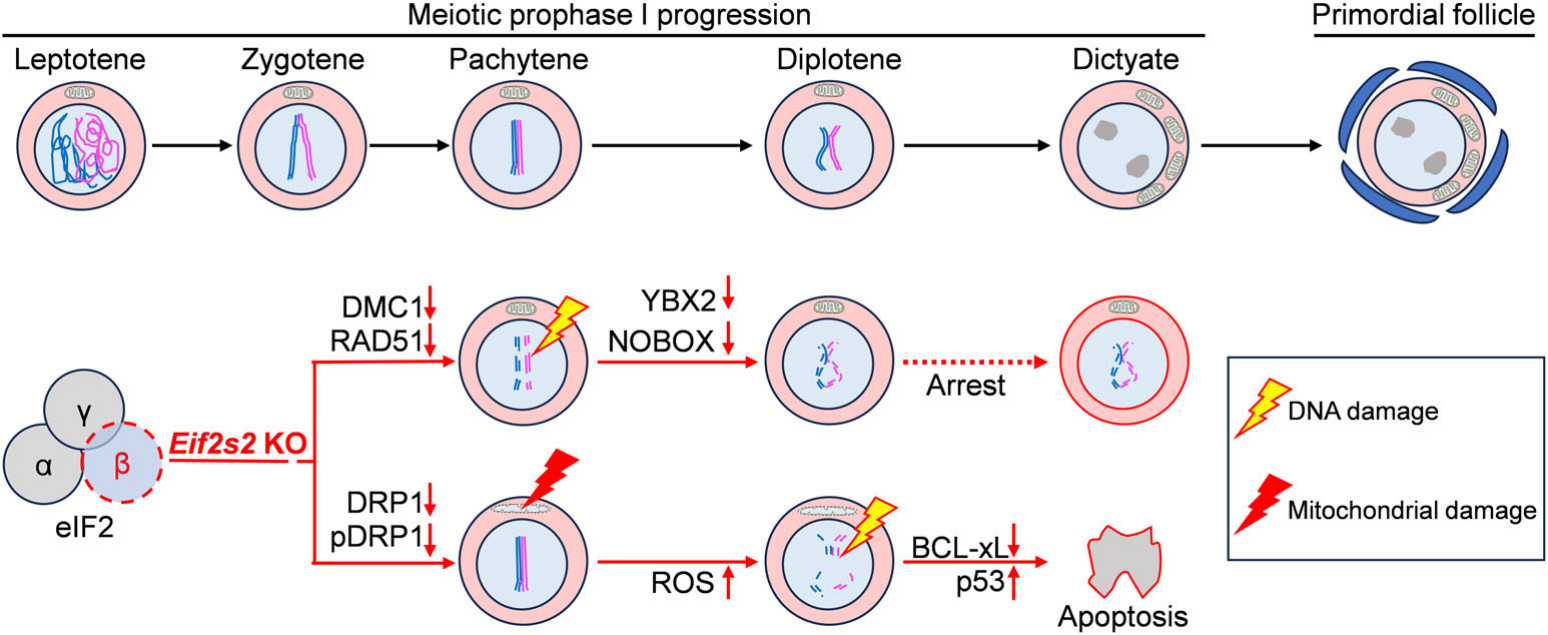
*Eif2s2* deletion in oocytes leads to failure of primordial follicle formation. Schematic summarizing the findings of this research. In general, normal oocytes will arrest at the dictyate stage and be enveloped by somatic cells to form primordial follicles. *Eif2s2* deletion impairing the translation of proteins related to homologous recombination and mitochondrial fission. These impairments resulted in oocyte meiotic arrest at the early diplotene stage, and eventually led to the apoptosis of oocytes and the failure of primordial follicle formation. Illustrations were created with ScienceSlides.

Mutations in translation initiation factors are closely associated with POI, impairing follicle development. This study demonstrates that the depletion of *Eif2s2* in premeiotic germ cells impairs the synthesis of HR‐related and mitochondrial fission‐related proteins in mouse oocytes. These impairments result in oocyte meiotic arrest at the early diplotene stage and ultimately led to the oocyte apoptosis and the failure of primordial follicle formation (Figure 11).

Deficiency in HR‐related proteins leaves DNA double‐strand breaks unrepaired, triggering the meiotic recombination checkpoint to cause meiotic arrest and apoptosis in mouse germ cells and yeasts.^28^, ^29^ In our study, the depletion of *Eif2s2* in premeiotic germ cells downregulated the crucial HR proteins DMC1 and RAD51 and then caused pachytene arrest in oocytes of *Eif2s2* ^*cKO*^ mice at 1 dpp, which may further lead to meiotic arrest at the early diplotene stage at 5 dpp. Consistently, the expression levels of dictyate genes were decreased in oocytes of *Eif2s2* ^*cKO*^ mice at 1dpp. Oocyte meiotic arrest at the early diplotene leads to the failure of primordial follicle formation.^30^ Furthermore, *Eif2s2* deletion caused abnormal mitochondrial fission, excessive ROS and DNA damage at 1 dpp, followed by oocyte apoptosis at 4 dpp. It is reported that abnormal mitochondrial fission compromises mitochondrial function and then induces excessive ROS in mouse and porcine GV‐stage oocytes.^31^, ^32^, ^33^ Excessive ROS accumulation can lead to oocyte apoptosis directly by decreasing the mitochondrial membrane potential and release of cytochrome c.^34^ Therefore, the oocyte apoptosis caused by the deletion of *Eif2s2* may be attributed to mitochondrial dysfunction‐induced ROS. On the other hand, excessive ROS can also lead to DNA damage in various cells,^35^, ^36^, ^37^ which will induce cell apoptosis via activating p53 and p63 signalling cascades.^38^, ^39^ *Dmc1*‐deficient mice exhibit a significant loss of oocytes at birth.^40^ However, the levels of HR protein were decreased, but no oocyte loss was observed in ovaries of *Eif2s2* ^*cKO*^ mice at 1 dpp. Therefore, depletion of *Eif2s2* in premeiotic germ cells causes oocyte apoptosis primarily by mitochondrial fission defect, ROS accumulation and subsequent DNA damage. Mutations in translation initiation factors commonly cause POI by impairing the follicular development in patients,^14^, ^15^ but our study demonstrates that the deficiency in translation initiation factor EIF2S2 could result in POI by diminishing the size of primordial follicle pool in newborn mice.

*Eif2s2* is crucial for protein translation that supports cell survival.^20^, ^21^ In our study, *Eif2s2* was deleted in germ cells in the mice at 12.5 days post‐coitum (dpc), but oocytes did not undergo apoptosis until 1 dpp. This could be attributed to the decrease in the ubiquitination levels in *Eif2s2* ^*cKO*^ mice. The deceleration of EIF2S2 and other protein degradation can maintain the protein translation initiation and oocyte survival, respectively. On the other hand, *Eif2s2* deletion induced the ISR, characterized by the downregulation of general protein translation and selective translation of ATF4 and its downstream proteins. The selective translation of *Atf4* mRNA can activate the pro‐survival response in human fibroblasts^41^ and HEK293T cells.^42^ Thus, the activation of ISR may also contribute to the survival of *Eif2s2*‐deleted oocytes. A recent clinical study reports that patients with Turner syndrome exhibit a lack of a cluster of oogonia with high *EIF2S2* expression and germ cell apoptosis at 12–13 weeks post‐conception, subsequently developing POI after birth.^43^ These findings suggest that insufficient expression of *EIF2S2* is associated with POI. Collectively, the aforementioned molecular mechanisms provide potential targets for the diagnosis and treatment of female infertility.

Our maternal stress mouse model revealed that maternal stress could decrease the number of oocytes and primordial follicles in newborn mice. The stressed mice showed higher levels of serum glucocorticoid CORT than those in the control group, which is consistent with previous research findings.^44^ In the present study, the injection of CORT activated ISR and induced oocyte apoptosis in newborn mice. This is consistent with a previous study showing that injection of another glucocorticoid cortisol induces oocyte apoptosis in adult mice.^45^ CORT has been demonstrated to induce ISR and apoptosis in neural cells.^46^, ^47^ ISRIB partially reversed detrimental effects of CORT on oocytes, consistent with other reports indicating that ISRIB attenuates the CORT‐induced apoptosis of neural cells by reversing the ISR‐induced upregulation of ATF4, and global translation repression.^48^, ^49^ Thus, our findings suggest that maternal stress reduces the size of the primordial follicle pool in newborn mice possibly by the elevating serum CORT levels and activating ISR in the ovary. The supplementation of CORT in the culture medium did not significantly affect primordial follicle formation in vitro, suggesting that mice exposed to various stresses may impair the primordial follicle formation via the hypothalamic‐pituitary‐adrenal (HPA) axis.^50^, ^51^, ^52^, ^53^, ^54^ Thus, in mammals, the safeguarding of pregnant females from stress is beneficial for the avoidance of the reduction in the size of the primordial follicle pool in their offspring.

In summary, our study has demonstrated that *Eif2s2* deletion in premeiotic germ cells causes oocyte meiotic arrest at the early diplotene stage and eventually leads to oocyte apoptosis and the failure of primordial follicle formation. Our study provides valuable insights into the diagnosis and treatment of female infertility.

## 4 MATERIALS AND METHODS

### 4.1 Animals and chemicals

WT C57BL/6 mice were purchased from the Guangdong Medical Laboratory Animal Center (Guangzhou, China). *Eif2s2* ^*fl/fl*^ mice (strain number T008925) were purchased from the GemPharmatech Co., Ltd. (Nanjing, China). To obtain oocyte‐specific *Eif2s2*‐deleted mice, exons 4 to 6 of *Eif2s2* were flanked by LoxP sites, and *Eif2s2* ^*fl/fl*^ mice were cross‐bred with *Stra8‐Cre* mice. Eventually, *Eif2s2* ^*fl/fl*^; *Stra8‐Cre* mice (*Eif2s2* ^*cKO*^ mice) were obtained by crossing *Eif2s2* ^*fl/+*^; *Stra8‐Cre* mice with *Eif2s2* ^*fl/fl*^ mice (Figure S1A). The primers utilized for genotyping are presented in Table S1. For restraint experiment, WT C57BL/6 mice were mated and pregnant mice at 16.5 dpc were divided into a control group and a restraint group. Each mouse in the restraint group was put into a 100 mL centrifuge tube with ventilation holes for 4 h/day for 3 days. The mouse in the centrifuge tube was able to move forward and backward. During the restraint treatment, mice from both control and restraint groups were deprived of food and water. Following delivery, the offspring were maintained with their parents for an additional 4 days. For injection experiments, newborn mice at 1 dpp were divided into control, CORT and CORT+ ISRIB groups. Mice from control, CORT and CORT+ISRIB groups were injected intraperitoneally with ethanol, 30 mg/kg CORT and 30 mg/kg CORT +2.5 mg/kg ISRIB, respectively. The CORT and ISRIB were prepared as stock solutions in ethanol. Mice from the control group were injected with the same concentration of ethanol. The animal protocols were approved by the South China University of Technology's Institutional Animal Care and Use Committee (IACUC, 2022102). All reagents were purchased from Sigma‐Aldrich (St. Louis, USA) unless stated otherwise.

### 4.2 Hormone assays

The blood samples of the control and restraint groups were collected and rested for 60 min at room temperature (RT). Then the blood samples were centrifuged (3000 rpm, 10 min) to separate serum. The serum collected was stored at −80°C for CORT assay. The serum CORT levels in control and restraint groups were quantified in accordance with instructions of the mouse CORT ELISA Kit (ELGBIO, EWE0544Mo).

### 4.3 Mouse ovary culture

The ovaries of newborn mice were harvested at the specified times. The procedure for mouse ovaries culture was described as previously.^55^ Briefly, mouse ovaries were separated and cultured on a membrane for 3 days in Dulbecco's modified Eagle's medium (DMEM)/F12 medium (1:1, supplemented with 1 mM pyruvate, 1% insulin–transferrin‐selenium and 3 mg/mL bovine serum albumin). The ovaries from newborn *Eif2s2* ^*fl/fl*^ and *Eif2s2* ^*cKO*^ mice were cultured in medium supplemented with 0 or 1 μM ISRIB. The ovaries of 1‐dpp WT C57BL/6 mice were cultured in medium supplemented with concentrations of 0, 10 or 50 μM CORT. CORT were prepared as stock solutions in dimethyl sulfoxide (DMSO). The control group received an equal concentration of DMSO. The concentration of DMSO did not exceed 0.1% in the culture system. The ovaries were maintained at 37°C with 5% CO_2_ and 100% humidity, and the medium was replaced every 2 days.

### 4.4 RNA isolation and quantification

Total RNA was isolated and purified from ovarian tissues as previously described.^56^ In brief, the RNA was extracted and reverse transcribed into cDNA. mRNA levels were quantified via qRT–PCR using SYBR Green PCR SuperMix (TransGen Biotech), which was performed on the Light Cycler 96 system (Roche, Basel, Switzerland). The relative expression levels of the target genes were calculated using the 2^−ΔΔCt^ method, with the ribosomal protein L19 (*Rpl19*) gene serving as the internal control. Additionally, for RNA‐seq analysis, ovaries from 1‐dpp *Eif2s2* ^*fl/fl*^ and *Eif2s2* ^*cKO*^ mice were collected. The RNA extracted from these samples was sent for analysis to Gene Denovo Biotechnology Co., Ltd (Guangzhou, China). The specific qRT–PCR primers utilized are detailed in Table S1.

### 4.5 Proteome analysis

The ovaries from 1dpp *Eif2s2* ^*fl/fl*^ and *Eif2s2* ^*cKO*^ mice were homogenized with lysis buffer (2% SDS, 7 M urea, 1 mg/mL protease inhibitor cocktail) for 3 min on ice using an ultrasonic homogenizer. After centrifugation at 15,000 rpm for 15 min at 4°C, the supernatant was collected and its protein concentration was determined using a BCA protein assay kit (Beyotime, China). The supernatant was then diluted to 1 μg/μL and incubated with 0.02 M dithiothreitol (Promega, USA) at 55°C for 1 h. Subsequently, 5 μL of 1 M iodoacetamide (Promega, Madison, WI) was added to 50 μL of the diluted supernatant to initiate protein precipitation. After overnight precipitation with 300 μL of precooled acetone at −20°C and redissolution in 50 mM ammonium bicarbonate, the proteins were digested with trypsin (Promega, USA) at a 50:1 (w/w) substrate/enzyme ratio at 37°C for 16 h. Peptide analysis was conducted using an Orbitrap Fusion Lumos coupled with an EASYnLC 1200 system (Thermo Fisher Scientific, USA) through a C18 analytical reverse‐phase column at 200 nL/min and 40°C. The mass spectrometer operated in data‐independent acquisition mode, switching automatically between MS and MS/MS modes. The raw data from the data‐independent acquisition (DIA) were processed and analysed using Spectronaut X (Biognosys AG, Switzerland) with the default settings. All experimental procedures and data analyses were conducted by Gene Denovo Biotechnology Co. Ltd. (Guangzhou, China).

### 4.6 Single cell isolation and scRNA‐seq

Ovarian cells were isolated and purified as previously described.^57^ After sorting in PBS containing 0.04% BSA, the cells were quantified and their integrity assessed using trypan blue and a Countess II automated counter (Thermo Fisher Scientific, USA). Cells were then loaded onto a 10x Genomics GemCode single‐cell instrument to produce single‐cell Gel Bead‐In‐Emulsion (GEM). The reverse transcription and library preparation were carried out using the Chromium Next GEM Single cell 3′ Reagent Kits v3.1 (10x Genomics, CG000204) according to the manufacturer's instructions. Sequencing of the library was performed using the Illumina NovaSeq 6000 system.

### 4.7 scRNA‐seq data preprocessing and analysis

The conversion of raw BCL files from the 10x Genomics scRNA‐seq platform into FASTQ files was accomplished using the Cell Ranger package (10x Genomics) to demultiplex and align the files to the mm10 transcriptome. Quality control was conducted using R software (v.4.1.0) and the Seurat package (v.4.0.6) (http://satijalab.org/seurat), marking cells as outliers based on gene count, UMIs and mitochondrial gene percentage, and excluding them from further analysis. Post‐filtering, 17,457 cells (8762 from *Eif2s2* ^*fl/fl*^ mice, 8695 from *Eif2s2* ^*cKO*^ mice) were available for downstream analysis. UMAP clustering was executed using the Seurat R package, applying the top 20 principal components after PCA on the top 2000 variable genes. The FindCluster function on a K‐nearest neighbor (KNN) graph model was used for clustering the cells with specified granularity. The FindAllMarkers function in Seurat identified the marker genes for each significant cluster. Cell types were ascertained using marker genes, supplemented by previous literature and the CellMarker database (http://bio-bigdata.hrbmu.edu.cn/CellMarker/). Oocyte cluster cells were selected and re‐clustered for further study. Graph‐based cluster identification and dimensionality reduction were achieved using DoHeatmap, FeaturePlot and DotPlot in Seurat to produce modularity heatmaps, UMAP plots and dot plots for cell‐type specific marker genes. Seurat was also employed to visualize gene expression individually by categorizing cells according to type, and to display the percentage of cells in each cluster and the scaled average gene expression for each cell type as a bar graph. Gene set enrichment on differentially expressed genes (DEGs) between *Eif2s2* ^*fl/fl*^ and *Eif2s2* ^*cKO*^ groups in the mast cell cluster was analysed and visualized using Metascape software (http://metascape.org/gp/#/main/step1). CellChat R package (1.3.0)^58^ was used to assess cell–to–cell interactions between oocytes and pregranulosa/epithelial cell clusters as previously described.^59^

### 4.8 Western blotting

Approximately 6–8 ovaries per replicate were collected for protein extraction. Total proteins were extracted as previously described.^60^ After separation of equivalent amounts of protein (10 μg) by SDS‐PAGE and transfer to a PVDF membrane (Millipore, USA), the membranes were blocked with 5% skimmed milk in Tris buffered saline with 0.5% Tween 20 (TBST) buffer. The membranes were incubated overnight at 4°C with primary antibodies (Table S2). After washing with TBST buffer, they were then treated with secondary antibodies (1:5000, Zhongshan Golden Bridge Biotechnology, China) for 1 h at RT. Bands were detected using the SuperSignal West Pico Kit (Thermo Fisher Scientific) and visualized using the Tanon 5200 chemiluminescent imaging system (Tanon, China). The relative intensities of the bands were quantified with ImageJ software (NIH Image, USA), using β‐Actin as an internal control. Uncropped scans of the representative blots are provided in the Supplementary materials (Figure S9).

### 4.9 Puromycin incorporation

Ovaries were treated with 1 μg/mL puromycin for 1 h at 37°C and collected after washing three times with PBS. Cell lysates were prepared and Western blot analysis was performed as described above. Puromycin signals were detected with anti‐puromycin primary antibody. The intensity of an electrophoretic lane indicates the amount of nascent polypeptides.

### 4.10 Immunofluorescence and ovarian histology assays

Ovaries from fetal or postnatal mice were fixed in 4% paraformaldehyde (PFA, Solarbio, P1110), embedded in paraffin and sectioned at 5 μm. These sections were deparaffinized, rehydrated and antigen retrieved in 0.01 M sodium citrate buffer (pH 6.0). After blocking with 10% donkey serum in PBS, they were incubated with primary antibodies (Table S2) and Alexa Fluor 488/555‐conjugated secondary antibodies (1:200, Thermo Fisher Scientific), followed by counterstaining with DAPI. For microtubule and γH2Ax visualization, ovaries were fixed in 4% PFA, treated as described above, and incubated with Alexa Fluor 488‐conjugated anti‐α‐tubulin antibody (1:200, Abcam) or Alexa Fluor 555‐conjugated anti‐γH2Ax antibody (1:300, Abcam) for 4 h at RT before DAPI counterstaining. Digital imaging was performed with a Zeiss LSM 800 confocal microscope (Carl Zeiss), and fluorescence intensity was quantified using ImageJ software, maintaining consistent exposure times across control and mutant/treatment groups. For histological studies, paraffin‐embedded ovaries were sectioned at 5 μm and stained with periodic acid/Schiff (PAS) reagent (only for 70 dpp ovary sections) and haematoxylin (Solarbio, China). Follicles with clearly visible nuclei, including primordial, growing and atretic follicles, were counted.

### 4.11 Meiotic surface spreading

Oocyte isolation was performed as described previously.^30^ Mouse ovaries were isolated and digested with 40 μL of TrypLE (Thermo, US) at 37°C for 10 min with continuous shaking. Digestion was halted by the addition of 4 μL fetal bovine serum (Beyotime, China). After a brief centrifugation at 1000 rpm for 1 min, the supernatant was removed, and the cells were processed with 20 μL of fixation buffer (1% paraformaldehyde, pH 9.2, with 10 mM dithiothreitol) containing 0.15% Triton X‐100. Subsequently, 20 μL aliquots of the cell suspension were placed on slides and allowed to dry overnight at RT. Immunofluorescence staining was then carried out as described above.

### 4.12 In situ cell death detection

Ovarian sections from *Eif2s2* ^*fl/fl*^ and *Eif2s2* ^*cKO*^ mice were analysed using the Click‐iT Plus TUNEL (terminal deoxynucleotidyl transferase‐mediated deoxyuridine triphosphate) assay kit (Thermo Fisher Scientific, USA). Briefly, deparaffinized sections were fixed in 4% PFA for 15 min, then permeabilized with proteinase K for 30 min. The sections underwent incorporation with EdUTP by incubation in the TdT reaction mixture for 60 min and were labelled with Alexa Fluor 488 dyes using the Click‐iT™ Plus TUNEL reaction cocktail in the dark for 60 min. Following a 5‐min incubation in 3% bovine serum albumin (BSA), the sections were counterstained with DAPI for 5 min and examined by a Zeiss LSM 800 confocal microscope (Carl Zeiss, Germany).

### 4.13 Transmission electron microscopy (TEM)

The TEM assay was conducted as previously described.^61^ In brief, ovaries were collected, cut into approximately 1 mm^3^ blocks and fixed in a fixative buffer (2.5% glutaraldehyde) for 12 h at 4°C. Then, the samples were dehydrated and sliced. The sections were stained with uranyl acetate and observed under a TEM (Hitachi, Japan).

### 4.14 Evaluation of reactive oxygen species and mitochondrial superoxide ROS production

The levels of ROS and mitochondrial superoxide in oocytes were detected according to the instructions. To detect ROS or mitochondrial ROS (mtROS), oocytes were stained in M2 medium with 10 μM 2′,7′‐dichlorodihydrofluorescein diacetate (S0033S, Beyotime) or 5 μM MitoSOX Red (RM02822, ABclonal) for 30 min at 37°C and then washed three times in PBS. Images were captured with an LSM800 confocal microscopy (Carl Zeiss, Germany) using the same parameters. The fluorescence intensity of each oocyte was quantified using ImageJ software.

### 4.15 Statistical analysis

Each experiment was repeated at least three times. We analysed and graphed the data using GraphPad Prism software (version 8.3.0, USA). The statistical significance between two groups was assessed with two‐tailed unpaired Student's *t*‐tests (*p < 0.05, **p < 0.01, ***p < 0.001).

## AUTHOR CONTRIBUTIONS

W.Z. and B.L. contributed equally to this work. W.Z., B.L. and M.Z. designed and performed the experiments. W.Z. analysed the data and wrote the manuscripts. B.L. and W.W. participated in the isolation of mouse ovaries. W.Z., B.L. and X.Z. participated in the follicle counting. S.H., Y.C. and X.Z. participated in genotyping of mice. W.Z. participated in mRNA detection and Western blotting. M.Z. conceived the idea and revised the manuscript. Every author has read and approved the final manuscript.

## CONFLICT OF INTEREST STATEMENT

The authors have declared that no competing interest exists.

## Supporting information


**Data S1.** Supporting information.

## Data Availability

RNA‐seq data have been submitted to the NCBI Sequence Read Archive (SRA) under accession number PRJNA1097299. Mass spectrometry proteomics data have been submitted to the ProteomeXchange Consortium via the iProX partner repository with the dataset identifier PXD051354. scRNA‐seq data have been submitted to the NCBI Gene Expression Omnibus (GEO) under accession number GSE263836. All data supporting the findings of this study are included within the article and its supplementary materials. Additional data related to this paper can be obtained upon request from the authors.
