# Supplementary material for: Premeiotic deletion of Eif2s2 causes oocyte arrest at the early diplotene stage and apoptosis in mice
Source: Cell Prolif. 2024 Jul 24;57(12):e13718. doi: 10.1111/cpr.13718 (PMC11628728; doi:10.1111/cpr.13718)
Supplement: Supplementary file 1 — Data S1. Supporting information. [file CPR-57-e13718-s001.docx]

Supplementary Material

**Pre-meiotic deletion of *Eif2s2* causes oocyte arrest at the early diplotene stage and apoptosis in mice**

Wenjun Zhou^1^, Biao Li^2^, Zhijuan Wang^1^, Shuang Liu^1^, Weiyong Wang^1^, Sihui He^1^, Ye Chen^1^, Xiaodan Zhang^1^, Meijia Zhang^1#^

1. The Innovation Centre of Ministry of Education for Development and Diseases, the Second Affiliated Hospital, School of Medicine, South China University of Technology, Guangzhou 510006, China

2. Center for Sleep and Circadian Medicine, The Affiliated Brain Hospital of Guangzhou Medical University, 36 Mingxin Road, 510370, Guangzhou, China.

^#^Corresponding author: Meijia Zhang, E-mail: zhangmeijia@scut.edu.cn


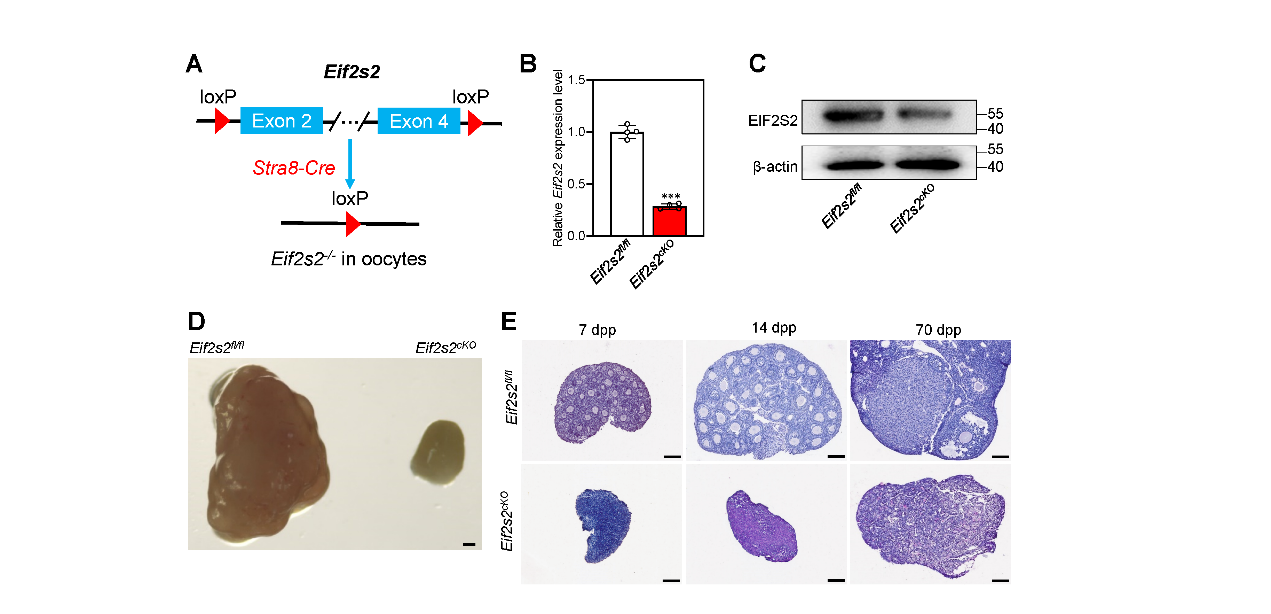


**Fig. S1 *Eif2s2* depletion in oocytes causes rapid oocyte loss. A** Schematic representation of the deletion of *Eif2s2* exon 2 to 4 in oocytes using Stra8-Cre. **B** qRT-PCR assay showed that *Eif2s2* mRNA level significantly decreased in ovaries of *Eif2s2^cKO^* mouse at 1dpp. **C** Western blotting analysis of EIF2S2 and β-actin expression within *Eif2s2^fl/fl^* and *Eif2s2^cKO^* mouse ovaries. **D** Photographs of ovaries of 70 dpp *Eif2s2^fl/fl^* and *Eif2s2^cKO^* mice. Scale bar: 200 μM. **E** Morphological comparison of ovaries from *Eif2s2^fl/fl^* and *Eif2s2^cKO^* mice at 7 dpp, 14 dpp and 70 dpp. Nuclei was stained by hematoxylin.


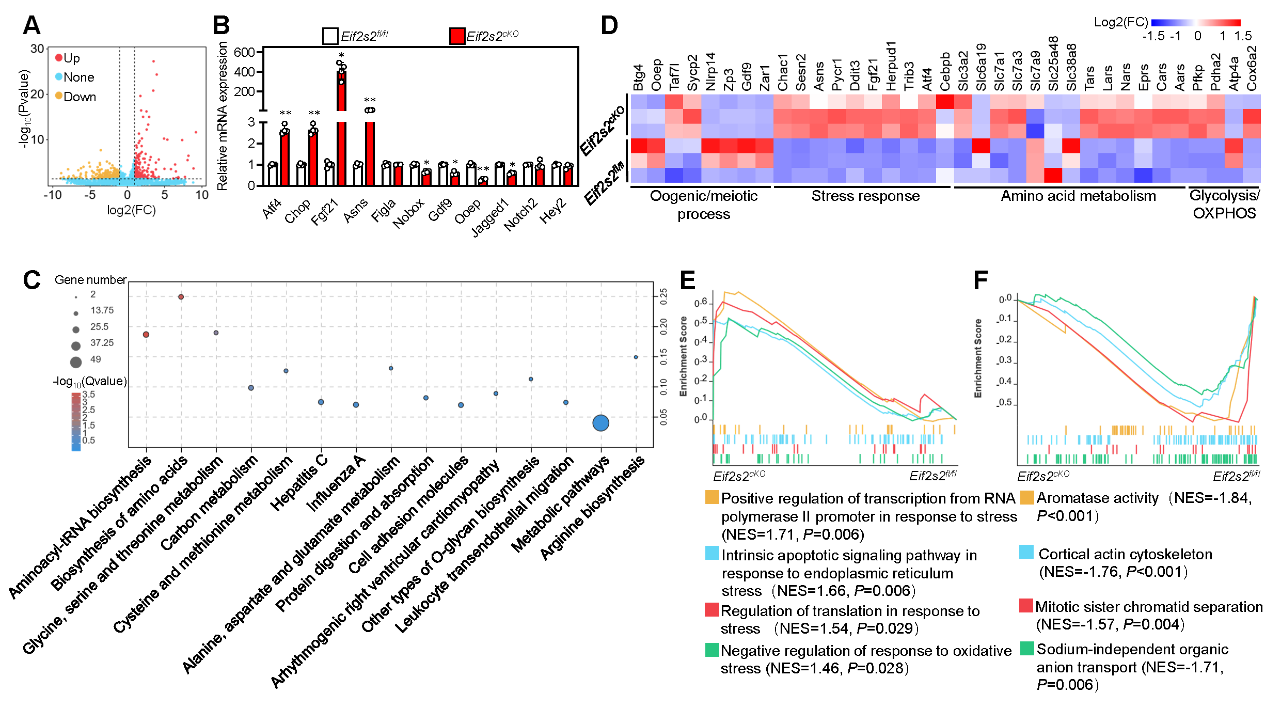


**Fig. S2 Transcriptome analysis of ovaries from *Eif2s2^fl/fl^* and *Eif2s2^cKO^* mice. A** Volcano plot comparing the transcripts of *Eif2s2^fl/fl^* and *Eif2s2^cKO^* ovaries at 1 dpp. Transcripts that increased or decreased by > 1.2-fold in *Eif2s2^cKO^* ovaries are highlighted in red or orange, respectively. **B** The mRNA levels of *Atf4*, *Ddit3*, *Fgf21*, *Asns*, *Figla*, *Nobox*, *Gdf9*, *Ooep*, *Jagged1*, *Notch1* and *Hey2* in ovaries from *Eif2s2^fl/fl^* and *Eif2s2^cKO^* mice at 1dpp. **C** Dot plot of partial significantly enriched KEGG pathways. **D** Heatmaps illustrating differences between *Eif2s2^fl/fl^* and *Eif2s2^cKO^* ovaries in the expression of a group of transcripts involved in various processes. **E and F** Gene set enrichment analysis (GSEA) revealing the enrichment of terms in *Eif2s2^cKO^* ovaries relative to *Eif2s2^fl/fl^*. NES, normalized enrichment score. Bars indicate the mean ± SD. A two-sided Student’s t-test was performed to determine *P* values (**P* < 0.05, ***P* < 0.01, and ****P* < 0.001).


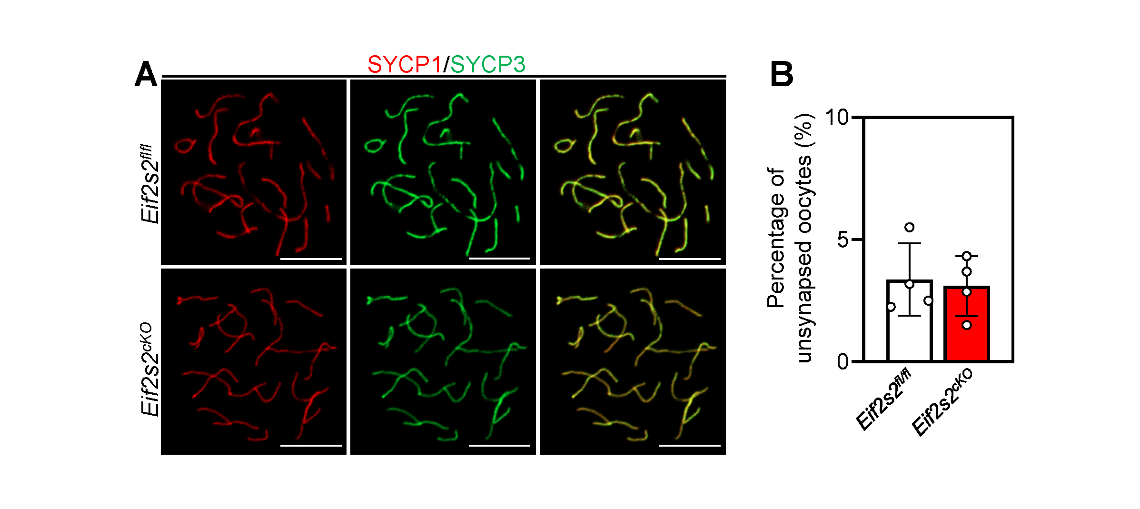


**Fig. S3 No differences are observed in percentage of oocytes with unsynapsed homologous chromosomes between** ***Eif2s2^fl/fl^* and *Eif2s2^cKO^* mice.** **A** Immunofluorescence staining of oocyte nuclear chromosome spreads by SYCP1 (red) and SYCP3 (green) markers in *Eif2s2^fl/fl^* and *Eif2s2^cKO^* mouse ovaries. Scale bar: 10 μM. **B** Statistical results showing the percentage of oocytes with unsynapsed homologous chromosomes in ovaries of *Eif2s2^fl/fl^* and *Eif2s2^cKO^* mice. n = 4. Bars indicate the mean ± SD. A two-sided Student’s t-test was performed to determine *P* values (**P* < 0.05, ***P* < 0.01, and ****P* < 0.001).


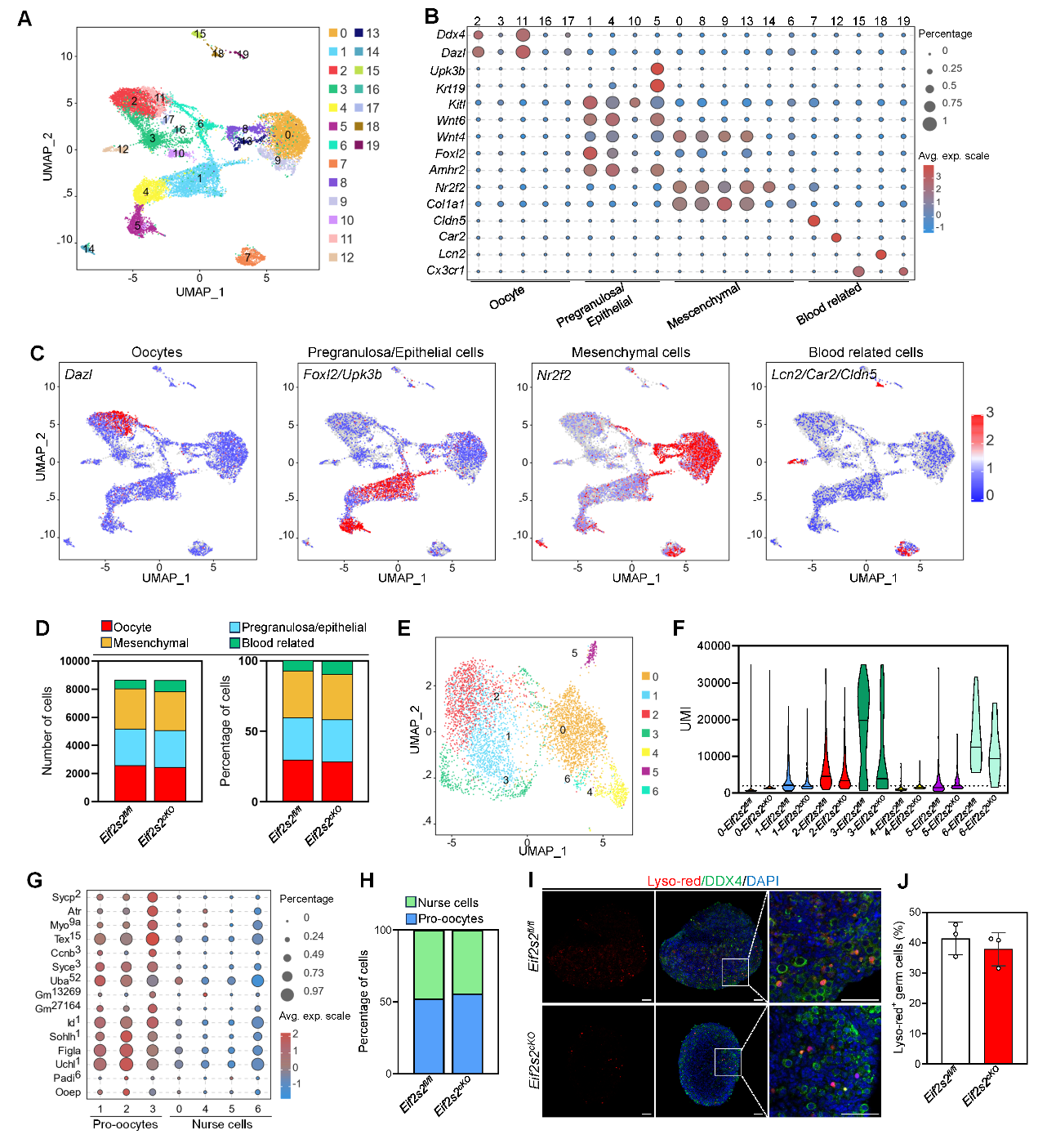


**Fig. S4 Single-cell RNA sequencing results reveal cell cluster types in ovaries and heterogeneity of germ cell subsets. A** uMAP plots of ovarian cells based on Seurat cluster. **B** Dot plot of marker genes identified from ovarian cell types in each cluster.

Dot size represents the percentage of gene expression in each cell cluster; color intensity refers to the average expression scale, with red color indicating high gene expression. **C** Feature plots of specific marker genes from 4 main ovarian cell types.

*Eif2s2^fl/fl^* and *Eif2s2^cKO^* mice. **D** Statistics on the number and percentage of different kinds of cells in ovaries from *Eif2s2^fl/fl^* and *Eif2s2^cKO^* mice. **E** and **F** The UMI/cell distributions of indicated clusters. **G** Dot plot of marker genes identified from different meiotic prophase one stages in each subcluster of oocytes. Dot size represents the percentage of gene expression in each cell cluster; color intensity refers to the average expression scale, with red color indicating high gene expression. **H** Statistics on the percentage of pro-oocytes and nurse cells in ovaries from *Eif2s2^fl/fl^* and *Eif2s2^cKO^* mice. **I** Representative images of staining of nurse cells using Lysotracker (Red). DDX4, Green. DAPI, Blue. **J** Statistics on the percentage of nurse cells in ovaries from *Eif2s2^fl/fl^* and *Eif2s2^cKO^* mice. Bars indicate the mean ± SD. A two-sided Student’s t-test was performed to determine *P* values (**P* < 0.05, ***P* < 0.01, and ****P* < 0.001).


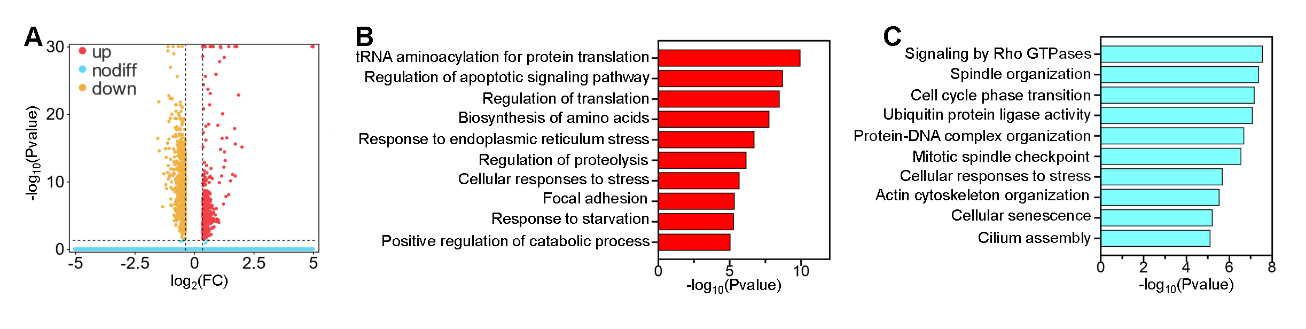


**Fig. S5 Enriched pathways of up regulated and downregulated genes in pro-oocyte of *Eif2s2^cKO^* mice. A** Volcano plot comparing the proteins of *Eif2s2^fl/fl^* and *Eif2s2^cKO^* pro-oocytes at 1 dpp. Transcripts that expressed at least more that 10% of cells and increased or decreased by > 1.283-fold in *Eif2s2^cKO^* pro-oocytes compared with *Eif2s2^fl/fl^* pro-oocytes are highlighted in red or orange, respectively. **B and C** Bar graphs illustrating the enriched GO/KEGG terms or canonical pathways associated with the transcripts that have a decreased (**B**) or an increased (**C**) level in *Eif2s2^cKO^* pro-oocytes.


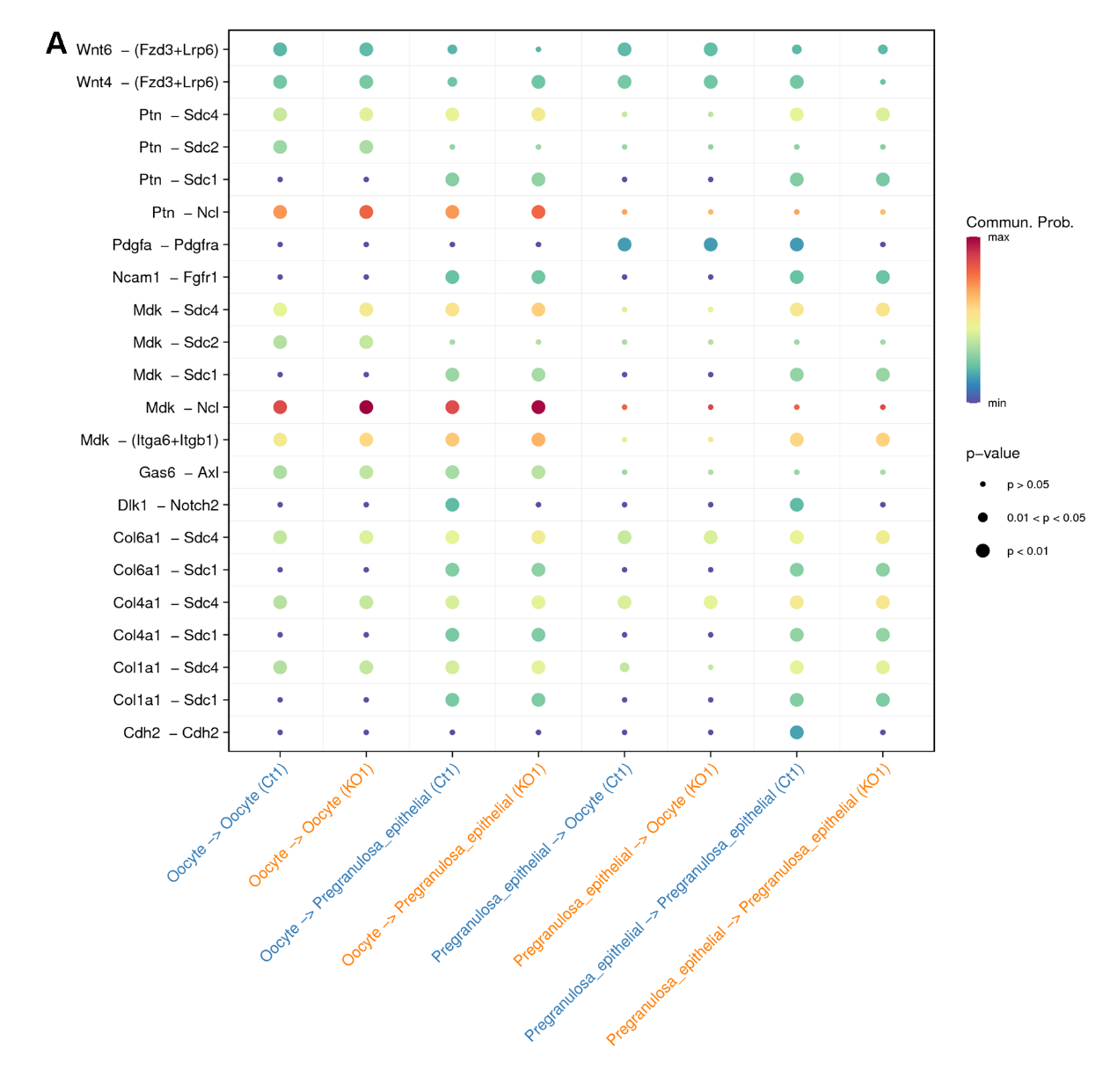


**Fig. S6 Cell-to-cell communication between oocytes and pregranulosa/epithelial cells in *Eif2s2^fl/fl^* and *Eif2s2^cKO^* mice. A** Dot plot of the expression levels of upregulated and downregulated signal ligand-receptor pairs in oocytes and pregranulosa/epithelial cells clusters of *Eif2s2^fl/fl^* and *Eif2s2^cKO^* mouse ovaries.


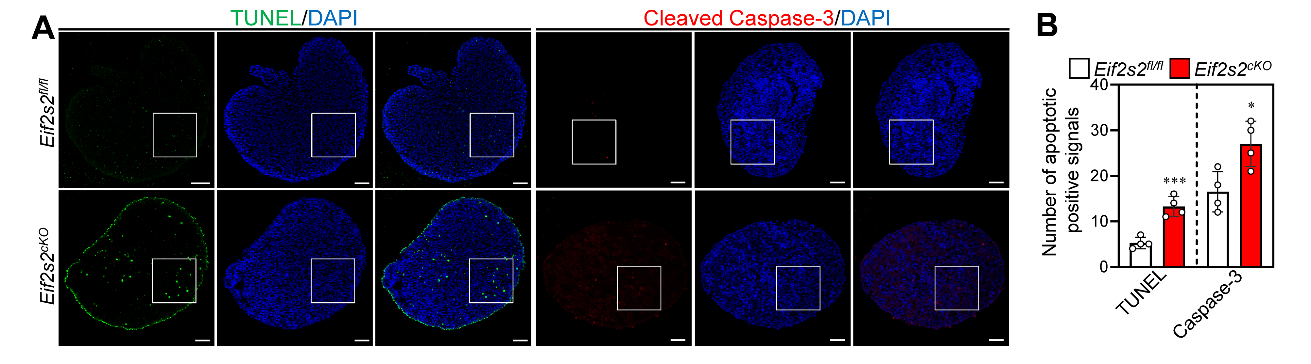


**Fig. S7** ***Eif2s2* deletion in oocytes causes increased apoptosis of cells.** **A** Immunostaining of TUNEL (green) and cleaved Caspase3 (red) in *Eif2s2^fl/fl^* and *Eif2s2^cKO^* mouse ovaries. DAPI, blue. Scale bar: 50 μM. **B** The number of cells with TUNEL- or cleaved-Caspase3-positive signals in *Eif2s2^fl/fl^* and *Eif2s2^cKO^* mouse ovaries. Bars indicate the mean ± SD. A two-sided Student’s t-test was performed to determine *P* values (**P* < 0.05, ***P* < 0.01, and ****P* < 0.001).


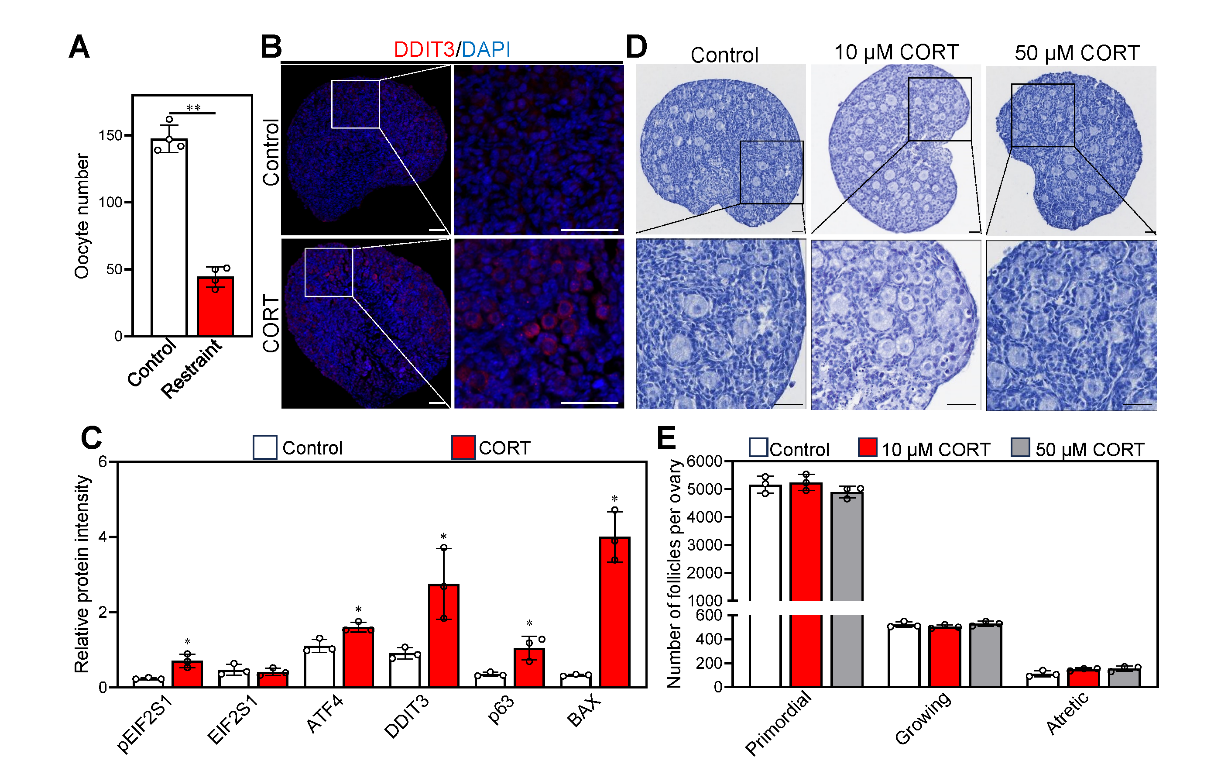


**Fig. S8 Stress-induced corticosterone activate integrated stress response in offspring’s ovaries. A** The number of oocytes in the control and restraint group mouse ovaries. n = 4. **B** Immunostaining of DDIT3 (red) in control and corticosterone (CORT) group mouse ovaries collected 48 h after injection. **C** Western blotting analysis of pEIF2S1, EIF2S1, ATF4, DDIT3, p63, and BAX expression in control and CORT group mouse ovaries at 48 h after CORT injection. β-actin was utilized as an internal control. **D and E** The ovaries were cultured in the medium supplemented with ethanol (control) or the medium supplemented with 10 and 50 μM CORT for 3 days. Morphological comparison of the ovaries (**D**) and the number of primordial, growing and atretic follicles (**E**) in different treatments. Nuclei were stained by hematoxylin. Scale bars: 50 μm. Bars indicate the mean ± SD. A two-sided Student’s t-test was performed to determine *P* values (**P* < 0.05, ***P* < 0.01, and ****P* < 0.001).


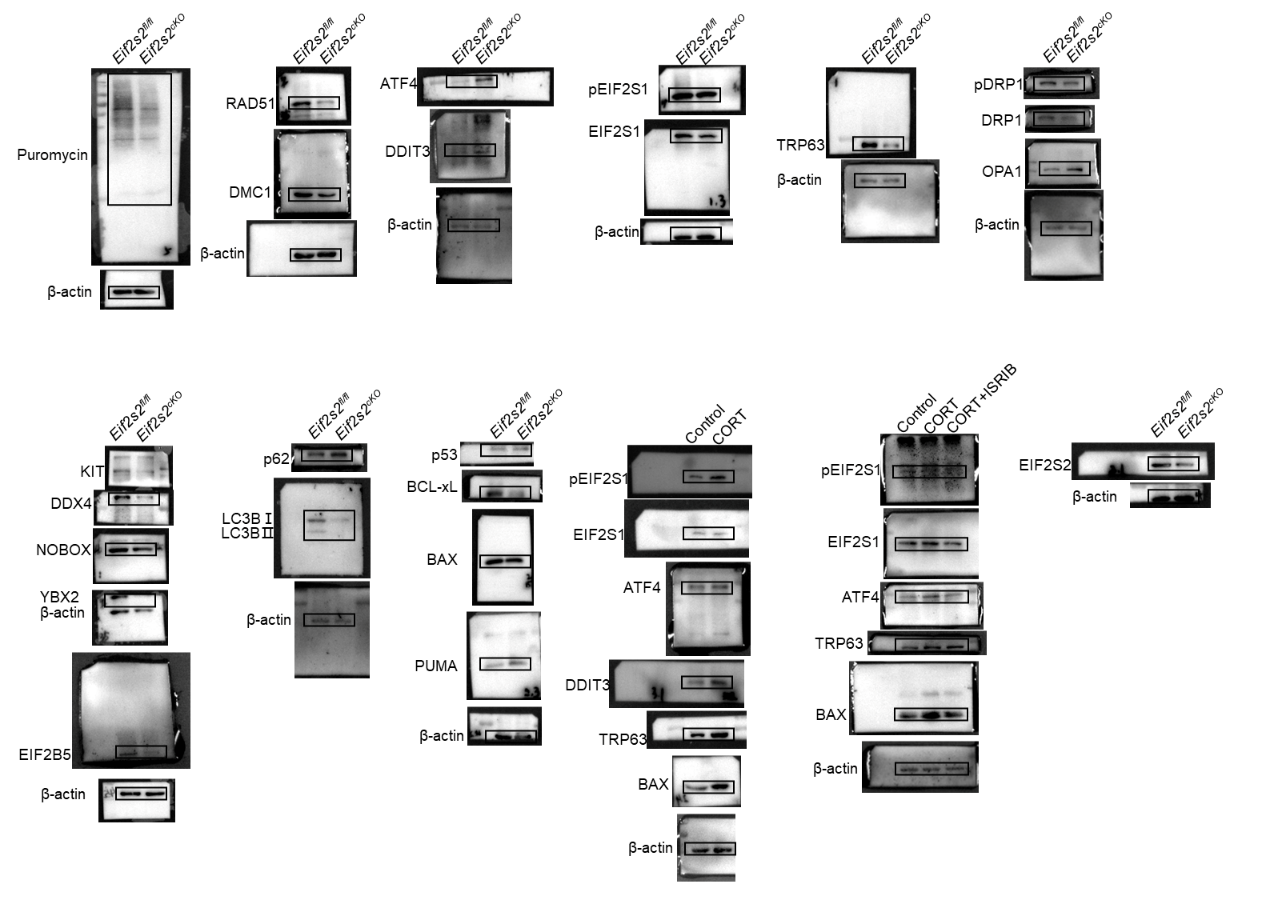


**Figure S9 Images of uncropped Western blot results.**

**Table S1** List of primers used in this study

|  | Forward primer (5'-3') | Reverse primer (5'-3') |  |
| --- | --- | --- | --- |
|  |  |  |  |
| *Eif2s2* | TGCTCCAATGTGCTTTCGATCA | GGAACCCTATCAGCAACCCTTAC | primers used in PCR for genotype identification of mice |
| *Stra8-Cre* | ACTCCAAGCACTGGGCAGAA | GCCACCATAGCAGCATCAAA |  |
|  |  | CGTTTACGTCGCCGTCCAG |  |
| *Figla* | GATACTCGGCTGTGTTCTGG | TGGTAGGTTGGGTAGCATTTC | primers used in qRT–PCR for detecting gene expression |
| *Nobox* | CTATCCTGACAGTGACAAACGCC | CACCCTCTCAGCACCCTCATTAT |  |
| *Ooep* | GTCATAGGCACAGACCAAGCG | GGCCGCCATGTTCAAGAGAAT |  |
| *Hey2* | CAAGGATCTGCCAAGTTAGAAAAG | TGTCAAGCACTCTCGGAATC |  |
| *Jagged1* | TGGACATTATGCCTGTGACC | CAACCGTACTGGCACCTG |  |
| *Atf4* | GCCTGACTCTGCTGCTT | TGGCTGCTGTCTTGTTT |  |
| *Asns* | GCAGTGTCTGAGTGCGATGAA | TCTTATCGGCTGCATTCCAAAC |  |
| *Fgf21* | CTGCTGGGGGTCTACCAAG | CTGCGCCTACCACTGTTCC |  |
| *Notch2* | GACCCTATCCTACCCTCTAGTG | AGCAGGATGAAGAACAGGATG |  |
| *Eif2s2* | GACATTGATGAAGCTGAAGAAGCTA | ATGTCAAGGTCATCCTCTGGC |  |
| *Chop* | AGCTGGAAGCCTGGTATGAGGA | AGCTAGGGACGCAGGGTCAA |  |

Table S2 List of primary antibodies used in immune detection.

| Antibody | Catalog Code | Source | Host | Dilution | |
| --- | --- | --- | --- | --- | --- |
|  |  |  |  | IF | WB |
| ATF4 | sc-390063 | Santa Cruz | Mouse |  | 1:1000 |
| BAX | 50599-2-Ig | Proteintech | Rabbit |  | 1:1000 |
| BCL-xL | 2764 | Cell Signaling Technology | Rabbit | 1:200 | 1:1000 |
| Cleaved Caspase-3 | 9664 | Cell Signaling Technology | Rabbit | 1:100 | 1:1000 |
| DDIT3 | 15204-1-AP | Proteintech | Rabbit | 1:200 | 1:1000 |
| DDX4 | ab27591 | Abcam | Mouse | 1:200 | 1:1000 |
| DMC1 | sc-373862 | Santa Cruz | Mouse |  | 1:500 |
| DRP1 | A2586 | ABclonal | Rabbit |  | 1:1000 |
| EIF2B5 | 29935-1-AP | Proteintech | Rabbit |  | 1:1000 |
| EIF2S1 | 11170-1-AP | Proteintech | Rabbit |  | 1:1000 |
| EIF2S2 | ab184549 | Abcam | Rabbit | 1:200 | 1:1000 |
| FOXL2 | NB100-1277 | Novus Biologicals | Goat | 1:300 |  |
| GM130 | ab52649 | Abcam | Rabbit | 1:200 |  |
| HORMAD1 | 13917-1-AP | Proteintech | Rabbit | 1:200 |  |
| KIT | AF1356 | R&D system | Mouse |  | 1:1000 |
| LC3B | NB100-2220 | Novus Biologicals | Rabbit | 1:200 | 1:1000 |
| Lamin B1 | HY-80205 | MCE | Rabbit | 1:200 |  |
| OPA1 | sc-393296 | Santa Cruz | Mouse | 1:50 |  |
| p62 | NBP1-48320 | Novus Biologicals | Rabbit |  | 1:2000 |
| p53 | ab26 | Abcam | Mouse |  | 1:1000 |
| TRP63 | #39692 | Cell Signaling Technology | Rabbit | 1:200 | 1:1000 |
| Phospho-DRP1 | AP1353 | ABclonal | Rabbit |  | 1:1000 |
| Phospho-eIF2α | #3398 | Cell Signaling Technology | Rabbit |  | 1:1000 |
| Puromycin | A23031 | Abclonal | Rabbit |  | 1:1000 |
| RAD51 | ab133534 | Abcam | Rabbit | 1:200 | 1:1000 |
| RPA2 | ab76420 | Abcam | Rabbit | 1:200 |  |
| SYCP1 | ab175191 | Abcam | Rabbit | 1:200 |  |
| SYCP3 | sc-74569 | Santa Cruz | Mouse | 1:100 |  |
| YBX2 | ab154829 | Abcam | Rabbit | 1:200 | 1:1000 |
| α-tubulin Alexa Fluor® 488 | ab195887 | Abcam | Mouse | 1:400 |  |
| β-actin | 4967 | Cell Signaling Technology | Rabbit |  | 1:1000 |
| γH2A.X Alexa Fluor® 555 | ab206900 | Abcam | Rabbit | 1:300 |  |

IF: Immunofluorescence WB: Western blotting
